# LINC00460/DHX9/IGF2BP2 complex promotes colorectal cancer proliferation and metastasis by mediating HMGA1 mRNA stability depending on m6A modification

**DOI:** 10.1186/s13046-021-01857-2

**Published:** 2021-02-01

**Authors:** Pingfu Hou, Sen Meng, Minle Li, Tian Lin, Sufang Chu, Zhongwei Li, Junnian Zheng, Yuming Gu, Jin Bai

**Affiliations:** 1 Cancer Institute, Xuzhou Medical University, 84 West Huaihai Road, Xuzhou, Jiangsu Province, 221002 China; 2 Center of Clinical Oncology, Affiliated Hospital of Xuzhou Medical University, Xuzhou, China; 3 Department of Interventional Radiography, Affiliated Hospital of Xuzhou Medical University, 99 West Huaihai Road, Xuzhou, Jiangsu Province, 221002 China

**Keywords:** LINC00460, Colorectal cancer, IGF2BP2, DHX9, HMGA1, m6A

## Abstract

**Background:**

Increasing studies have shown that long noncoding RNAs (lncRNAs) are pivotal regulators participating in carcinogenic progression and tumor metastasis in colorectal cancer (CRC). Although lncRNA long intergenic noncoding RNA 460 (LINC00460) has been reported in CRC, the role and molecular mechanism of LINC00460 in CRC progression still requires exploration.

**Methods:**

The expression levels of LINC00460 were analyzed by using a tissue microarray containing 498 CRC tissues and their corresponding non-tumor adjacent tissues. The correlations between the LINC00460 expression level and clinicopathological features were evaluated. The functional characterization of the role and molecular mechanism of LINC00460 in CRC was investigated through a series of in vitro and in vivo experiments.

**Results:**

LINC00460 expression was increased in human CRC, and high LINC00460 expression was correlated with poor five-year overall survival and disease-free survival. LINC00460 overexpression sufficiently induced the epithelial–mesenchymal transition and promoted tumor cell proliferation, migration, and invasion in vitro and tumor growth and metastasis in vivo. In addition, LINC00460 enhanced the protein expression of high-mobility group AT-hook 1 (HMGA1) by directly interacting with IGF2BP2 and DHX9 to bind the 3′ untranslated region (UTR) of HMGA1 mRNA and increased the stability of HMGA1 mRNA. In addition, the N6-methyladenosine (m6A) modification of HMGA1 mRNA by METTL3 enhanced HMGA1 expression in CRC. Finally, it suggested that HMGA1 was essential for LINC00460-induced cell proliferation, migration, and invasion.

**Conclusions:**

LINC00460 may be a novel oncogene of CRC through interacting with IGF2BP2 and DHX9 and bind to the m6A modified HMGA1 mRNA to enhance the HMGA1 mRNA stability. LINC00460 can serve as a promising predictive biomarker for the diagnosis and prognosis among patients with CRC.

**Supplementary Information:**

The online version contains supplementary material available at 10.1186/s13046-021-01857-2.

## Background

Long noncoding RNAs (lncRNAs) are a class of large transcripts with more than 200 nucleotides having no or limited protein-coding capacity, lncRNAs are pervasively transcribed from the human genome [1, 2]. With the unprecedented progress in understanding the function of lncRNAs, increasing evidence has shown that lncRNAs play a role in the physiological and pathological processes of various diseases, especially malignancies [3]. Although many findings should be further validated, lncRNAs can carry out diverse functions in carcinogenesis, metastasis, and poor prognosis in cancer [4, 5]. For example, the upregulated expression of CCAT2 in colorectal cancer (CRC) is correlated with migration and metastasis [6]. MALAT1 was identified as a prognostic biomarker that promotes cell proliferation and metastasis in non-small cell lung cancer (NSCLC) and other cancers [7]. Many lncRNAs and their underlying mechanisms in CRC have been previously reported [8, 9]. However, the specific mechanisms of lncRNAs in CRC development and metastasis should be further elucidated.

CRC is one of the most lethal malignancies worldwide and is also the leading cause of cancer incidence rate and mortality in China [10]. Most patients are diagnosed at the advanced stage and suffer from poor prognosis [11]; furthermore, malignant proliferation and extensive metastasis have already occurred in the advanced stage of CRC upon diagnosis [12]. Reports indicate that 50% of patients with CRC die from developing distant metastasis [13], and those with liver metastasis feature a short five-year survival rate of less than 10% [14]. The mechanism of colorectal carcinogenesis and metastasis is considered a multifactorial and multistep process that may involve several biological pathways and genetic alterations. Therefore, novel biomarkers that can promote or inhibit the development and progression of CRC should be sought.

LncRNAs are increasingly being discovered, and they may affect the complicated metastasis progress. In the present study, we assayed clinical specimens and adjacent normal tissues from patients with CRC to screen differentially expressed lncRNAs. We identified a novel lncRNA, called long intergenic noncoding RNA 460 (LINC00460), which is positively correlated with CRC proliferation and metastasis. LINC00460 is located on chromosome 13q33.2 and is transcribed into a 935 bp transcript; this lncRNA has recently been reported in several cancers. Most recently, several studies have shown LINC00460 was overexpressed in multiple cancers, such as gastric cancer [15], lung cancer [16], head and neck squamous cell carcinoma (HNSCC) [17], hepatocellular carcinoma [18] and colon cancer [19, 20]. Studies showed that LINC00460 mainly located in cytoplasm and could bind to miRNAs to affect CRC progression [19, 20]. A recent study showed LINC00460 interacted with PRDX1 and facilitated PRDX1 entry into the nucleus to promote HNSCC malignant properties [17]. However, its biological role and specific mechanism in CRC malignant proliferation and extensive metastasis should be further uncovered.

Most recently, Marco Mineo et al. have demonstrated that lncRNA HIF1A-AS2 binding proteins insulin-like growth factor 2 mRNA-binding protein 2 (IGF2BP2) and ATP-dependent RNA helicase A (DHX9) are engaged in downstream mRNA stabilization [21]. The High Mobility Group A1 (HMGA1) is a member of non-histone chromatin protein, and regulates multiple biology events, such as embryogenesis, proliferation, transformation, invasion, and metastasis [22]. Recently, studies have showed that HMGA1 can be regulated by IGF2BP2 through regulating HMGA1 mRNA stability [21, 23]. IGF2BP2 is also reported as a N6-methyladenosine (m^6^A) reader enhancing mRNA stability through recognizing m^6^A modification sites [24]. Methyltransferase-like 3 (METTL3) is a catalytic enzyme that promotes m^6^A modification of mRNAs [25]. The m^6^A modification in mRNAs have been shown regulating mRNA stability [26]. However, the m^6^A modification in regulating HMGA1 has not been reported.

In the present study, we showed that LINC00460 is highly expressed in CRC and indicates poor prognosis. We also revealed that LINC00460 regulated the CRC growth and metastasis in vitro and in vivo. LINC00460 interacted with IGF2BP2 and (DHX9) to promote the mRNA stability of HMGA1 and further leading to biological response to CRC malignant proliferation and extensive metastasis. In addition, the (m^6^A) modification of HMGA1 mRNA by METTL3 enhanced its expression in CRC and LINC00460 regulate HMGA1 expression depending on METTL3. It suggested LINC00460/DHX9/IGF2BP2 complex may regulate HMGA1 expression through recognizing the m6A modification sites of HMGA1 to enhance its mRNA stability.

## Methods

### Patients and sample collection

For this study, the tissue microarrays (TMAs) slides included examination of 498 pairs of tissue specimens including CRC tissues and the matched corresponding adjacent normal colorectal tissues from CRC patient cohorts that were enrolled at Affiliated Hospital of Xuzhou Medical University from 2010 to 2015 in China. Clinical and pathological information was obtained from the medical records of the Affiliated Hospital of Xuzhou Medical University. Survival time was calculated based on the date of surgery to the date of death or to the last follow-up. The patient studies were conducted in accordance with Declaration of Helsinki. The use of these specimens and data for research purposes were granted approval by the Ethics Committee of the Affiliated Hospital of Xuzhou Medical University.

### Cell culture and cell treatment

The CRC cell lines and HEK293T cell were obtained from the cell bank of Chinese academy of sciences. SW480, SW620, HCT116, DLD1, LOVO, HT29, FHC and HEK293T cells were cultured in 1640 and DMEM Medium supplemented with 10% fetal bovine serum, 100 U/ml penicillin, 100 μg/ml streptomycin, and incubated in a 37 °C humidified incubator with 5% CO_2_. 1% O_2_ was generated by flushing a 94% N_2_/5% CO_2_ mixture into the incubator.

The small interfering RNAs (siRNA, 50 nM) against human IGF2BP2 and DHX9 were transfected into the CRC cells SilenFect reagent (Thermo Fisher Scientific Inc., USA), while non-specific siRNA was used as negative controls. All siRNAs were purchased from Genepharma Technology (Shanghai, China). The sequences of the siRNAs were listed in below:

siIGF2BP2:

CAGUUUGAGAACUACUCCUTTAGGAGUAGUUCUCAAACUGTT.

siDHX9:

GCCUCCAAGAAAGUCCAAUTTAUUGGACUUUCUUGGAGGCTT.

### Establishment of stable cell lines

The cDNA of LINC00460 was inserted into pCDH-CMV-MCS-EF1-GreenPuro lentivirus vector at ECOR1/BamH1 sites. LINC00460 shRNA sequences were cloned to the vector pLko.1 at Age1/ECOR1 sites. The METTL3 shRNA vectors were purchased from GENE company (Shanghai, China). The overexpression and knockdown lentiviruses were generated by co-transfecting 293 T cells with the other two packing vectors pMD2G and psPAX. The supernatants of 293 T cultured medium were collected 48 h later, filtered through 0.45-mm filters (Millipore, Temecula, CA, USA) and concentrated using Amico Ultra centrifugal filters (Millipore 100KD MWCO). The concentrated virus was used to infect HCT116 and SW480 cells and stable LINC00460 overexpression or knockdown cells were generated by lentivirus infection. The relative LNC00460 and METTL3 shRNA sequences were described as below:

ShLINC00460#1-For: CCGGGGTACCCAGACATTGTTATGACTCGAGTCATAACAATGTCTGGGTACCTTTTTG;

ShLINC00460#2-For: CCGGGGAGGCGTCTGTGTAGCAATTCTCGAGAATTGCTACACAGACGCCTCCTTTTTG;

shMETTL3#1-For: CCGGGCAAGTATGTTCACTATGAAACTCGAGTTTCATAGTGAACATACTTGCTTTTTG.

shMETTL3#2-For:

CCGGGCCAAGGAACAATCCATTGTTCTCGAGAACAATGGATTGTTCCTTGGCTTTTTG.

### LncRNA microarray analysis

Total RNA was extracted from five paired CRC tissues and NCTs using TRIzol Reagent (Invitrogen, USA). RNA quantity and quality were measured by NanoDrop ND-1000. RNA integrity was assessed by standard denaturing agarose gel electrophoresis. LncRNA microarray was performed by (Aksomics, Shanghai, China). Arraystar Human LncRNA Microarray V3.0 is used for detecting the global profiling of human LncRNAs. Agilent Feature Extraction software (version 11.0.1.1) was used to analyze acquired array images. LncRNAs were deemed differentially expressed if their fold change between the CRC and NCT (normal colon tissue) groups exceed 2.0 and their *P*-values were less than 0.05.

### RNA extract, reverse transcription-PCR and qRT-PCR

RNA was extracted using TRIzol (Invitrogen) and cDNA was synthesized using the HiScript 1st Strand cDNA Synthesis Kit (Vazyme Biotech, Nanjing, China). Real-time PCR was carried out on ABI-7500 using UltraSYBR One Step RT-qPCR Kit (CWBIO, Beijing, China). The primers using for quantitative RT-PCR analysis were listed in Additional file 1: Table S1:

### Western blot and antibodies

Cells were harvested, total protein from cells was extracted by using RIPA lysis buffer and was qualified by using a BCA detecting kit (Keygen, Nanjing, China). Proteins samples were subjected to 7.5% or 10% SDS-PAGE and transferred onto a PVDF membrane, and then incubated with specific antibody at 4 °C overnight, respectively. The next day, the membranes were incubated with secondary antibodies included HRP-goat anti-mouse, HRP-goat anti-rabbit (ABclonal) at room temperature for 1 h. Protein bands were detected on Tanon 5200 automatic chemiluminescence imaging analysis system using ECL reagent (Tanon, Shanghai, China). Antibody against GAPDH was used as control (sc-32,233, Santa Cruz, Dallas, TX, USA). HMGA1 (A1635, ABclonal, Wuhan, China), IGF2BP2 (11601–1-AP, Proteintech, USA), DHX9 (17721–1-AP, Proteintech, USA), E-cadherin (610,181,BD Biosciences, Bedford, Massachusetts, USA), N-cadherin (610,920,BD Biosciences, Bedford, Massachusetts, USA),β-catenin (610,154, BD Biosciences, Bedford, Massachusetts, USA), were used for Western blot assays.

### In situ hybridization (ISH) and immunohistochemistry (IHC)

The in situ hybridization of LINC00460 was performed on formalinfixed paraffin-embedded sections using specific oligonucleotide probe (Hs-LINC00460, RNAscope® 2.5, ACD, US), following the manufacturer instructions. Positive control probe (Hs-PPIB) and Negative control probe (DapB) were included for each hybridization procedure and analyzed using an OLYMPUS microscope with OLYMPUS OlyVIA V2.9 software.

IHC assays was implemented following a standard streptavidin-peroxidase (SP) method as previously reported [27] and heat induced epitope retrieval (HIER) was performed with the retrieval buffer, citrate, pH 6.0, prior to commencing with IHC staining protocol. For primary antibody incubation, anti-HMGA1 (A1635, ABclonal, Wuhan, China) antibody at 1:100 dilution. The slide without primary antibody incubation served as negative control.

### RNA fluorescence in situ hybridization (FISH)

Cells were seeded on glass bottom cell culture dish (801,001, NEST) for about 24 h, then cells were fixed with 10% neutral buffer formalin, incubation with hydrogen peroxide (2,005,617, RNAscope®) and protease III (2,004,920, RNAscope®) for about 10 min separately. After cell preparation and pretreatment, following the manufacturer instructions and used specific oligonucleotide probe (RNAscope® 2.5, ACD, US) to visualize the single RNA in each cell. The nuclei were stained with DAPI for 15 min. Finally, images were taken under a confocal microscope.

### Cellular proliferation, colony formation assays

CCK-8 assay was applied to measure the cell proliferation according to the Cell Counting Kit-8 manufacturer protocol (Dojindo, Japan). Colony formation assay was performed in 60 mm dishes one thousand cells seeded and two weeks cultured. Colonies were counted manually after staining with 0.1% crystal violet.

### Real time cellular analysis (RTCA)

The Real Time Cell Analyzer (xCelligence, RTCA, USA) were used for examined the effect of LINC00460 on cell proliferation. The xCELLigence system was used according to the instructions of the supplier (ACEA Biosciences). The system measured impedance differences to derive cell index values at time points and it may be set by the operator.

The xCELLigence system consists of four main components: the RTCA analyzer, the RTCA DP station, the RTCA computer with integrated software and disposable E-plate 16. Firstly, the optimal seeding concentration for proliferation assay was determined. After seeding the number of 5000 cells in 200 ml medium to each well in E-plate 16, the attachment and proliferation of the cells were monitored every 15 min. All experiments were carried out for 96 h .

### Cell migration, invasion and wound healing assays

Cells were starved in serum-free media for 24 h then added to the top chamber of 24-well transwell chambers plates (8.0 μm, Corning, NY, USA.). Specially, for the invasion assay, cells were seeded into the top chamber coated with Matrigel (BD Biosciences). Complete medium was added to the bottom chambers to stimulate migration or invasion. After incubation for 24–48 h, the cells those adhered to the lower surface of the membrane were stained with 0.1% Crystal Violet, and then the percentages of migrated or invasion cells were calculated. Five randomly selected fields per filter were counted.

In wound healing assay, cells were seeded at a density of 1 × 10^6^ cell/well onto six-well plates and cultured to about 80% confluence. Then, a sterile 10-μl pipette tip was used to form artificial scratches for each well. The suspended cells were washed away with PBS, and then the cells were cultured in medium with 1% FBS. Cell migration distance was photographed at 0 h and 24 h under an inverted light microscope.

### RNA pull-down assay and mass spectrometry

RNA pull-down assay was carried out as described briefly: in vitro biotin-labeled RNAs (LINC00460 and the antisense RNA) were transcribed with Biotin RNA Labeling Mix (Promega Corporation, US) and T7 RNA polymerase (Thermo Fisher Scientific, US) treated with RNase inhibitor, and purified with Clean-up kit (Promega Corporation, US). The biotinylated LINC00460 probes were dissolved in binding and washing buffer and incubated with Streptavidin Agarose Resin (Thermo Fisher Scientific, US). Then cell lysates of HCT116 and SW480 were incubated with probes-coated streptavidin beads, and pull-down proteins were run on SDS-PAGE gels, and then gels were stained by Coomassie Blue staining, and differential bands were cut off for Mass spectrometry (Shanghai Applied Protein Technology Co., Ltd. China).

### RNA immunoprecipitation

The RIP experiment was carried out with the EZ-Magna RIP Kit (Millipore) according to the manufacturer’s protocol using 5 mg of antibody. HCT116 and SW480 cells were lysed in complete RIP lysis buffer, and the cell extract was incubated with protein A/G agarose beads conjugated with specific antibodies or control IgG for 2 h at 4 °C. Beads were washed and incubated with Proteinase K to remove proteins. Finally, purified RNA was subjected to quantitative RT-PCR analysis. Antibody IGF2BP2 (11601–1-AP, Proteintech, China), DHX9 (17721–1-AP, Proteintech, China).

#### MeRIP

MeRIP, we used Anti-N6-methyladenosine (m6A) antibody (ab151230) to pull down m6A modified HMGA1. Total RNA was extracted from cells using Trizol (Thermo-Fisher). 100 μg RNA was added to 500 μl MeRIP buffer (150 mM NaCl, 10 mM Tris-HCl, pH 7.5, 0.1% NP-40) and incubated briefly with 1 μl rabbit IgG and then the IgG was removed by protein A/G beads. The pre-cleaned lysates were transferred to new tubes and incubated with rabbit IgG or m6A antibody for 2 h at 4 °C with rotation. Finally, m6A bound RNA was extracted with Trizol and the RNA level of HMGA1 was measured by qRT-PCR.

#### Animal work

The female BALB/c nude mice (6–8 weeks old) were purchased from Beijing Vital River Laboratory Animal Technology Co., Ltd. (Beijing, China). All animal experiments were approved by the Animal Care and Use Committee at Xuzhou Medical University. Groups of HCT116-Luc-shCtrl, HCT116-Luc-shLINC00460, and HCT116-Luc-shLINC00460 + HMGA1 cells (5 × 10^6^) were injected subcutaneously into the flanks of mice correspondingly. Tumors volume (V) was monitored every 2 days by measuring the long axis (L) and the short axis (W) of xenograft tumor and calculated with the following formula: V = (L × W^2^)/2.

### Bioinformatics of gene expression database

The correlation of LINC00460 expression and CRC patient survival were analyzed using the datasets generated with Affymetrix HGU133 Plus 2.0 microarrays. Data are deposited at the Gene Expression Omnibus (GSE40967). GSE40967 contains 566 CRC tissues.

### Statistical analysis

Statistical analyses were carried out using SPSS 20.0 software (SPSS Inc., Chicago, IL, USA) and GraphPad Prism 8 The association between LINC00460 and the clinicopathologic parameters of the CRC patients were evaluated by a Chi-square test. The Kaplan–Meier method and log-rank test were used to evaluate the correlation between LINC0046 expression and CRC patient survival. The unpaired t test was used to determine the statistical significance of differences between groups. Data were presented as mean ± SD. *p* < 0.05 was considered statistically significant.

## Results

### Overexpression of LINC00460 occurs in CRC and correlates with poor prognosis

**Fig. 1:**
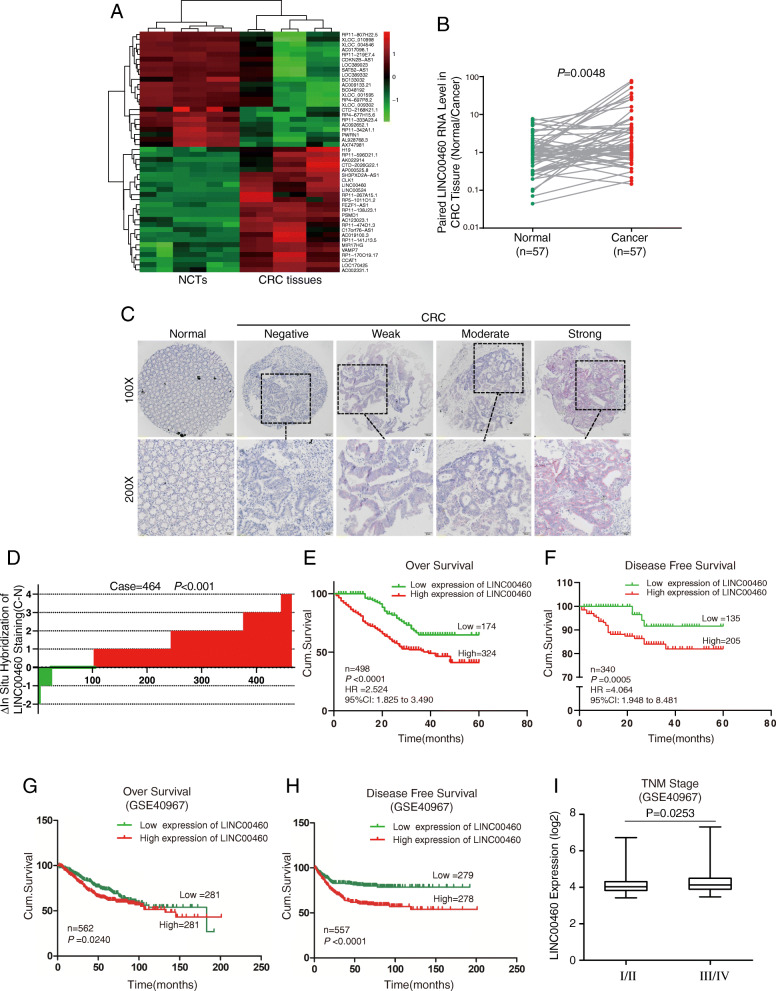
Overexpression of LINC00460 is observed in CRC and correlates with poor prognosis. **a** Heatmap of top 50 dysregulated lncRNAs identified from microarray analysis. NCTs: Normal colon tissues. **b** LINC00460 expression was quantified in colorectal cancer tissues and their adjacent non-cancerous tissues in a small cohort (*n* = 57, *p* = 0.0048). **b** LINC00460 expression was validated in 8 pairs of CRC patient samples by PCR and gel electrophoresis, N, normal tissue; T, tumor. **c** representative images of LINC00460 expression in colorectal cancer and adjacent normal tissues using ISH analysis in large cohort (*n* = 498). **d** Staining intensities of LINC00460 in colorectal cancer tissues compared with paired adjacent non-cancerous tissue. N, paired adjacent non-cancerous tissues. C, colorectal cancer tissues (*n* = 464, *p* < 0.001). **e**, **f** Kaplan–Meier survival curves depicting overall survival (n = 498, *p* < 0.0001) or disease-specific survival of patients with CRC (*n* = 340, p < 0.001) stratified by LINC00460 expression levels in CRC tissues. **g**, **h**, **i** The GEO date of LINC00460 expression was in CRC correlated with poor survival in over survival, disease-free survival, and advanced tumor stage (GSE40967)

LncRNA expression profiles were analyzed in six paired CRC tissues and normal colorectal tissues (NCTs) to screen differentially expressed lncRNAs by using lncRNA microaaray. Among the lncRNAs tested in our microarray, 462 were significantly upregulated, and 817 were significantly downregulated in CRC relative to NCT (fold change> 2, *p* < 0.05, Additional file 2: Dataset 1). Among the 50 lncRNAs showing the greatest changes, some known oncogenic lncRNAs in CRC, such as FEZF1-AS1, CCAT1, and H19, were found. The novel lncRNA LINC00460 was one of the most expressed lncRNAs (Fig. 1a). Thus, we focused on LINC00460 for further studies. Expressions of LINC00460 were validated in a small CRC cohort, revealing that LINC00460 was upregulated in CRC tissues relative to adjacent NCTs, as shown in RT-PCR (*p* = 0.0048, Fig. 1b) and gel electrophoresis results (Additional file 3: Figure S1). LINC00460 expression was examined via in situ hybridization (ISH, RNAscope®) in an expanded CRC cohort. The ISH arrays showed that LINC00460 was overexpressed in CRC tissues compared with the adjacent NCTs (*p* < 0.001, Fig. 1c–d). ISH analysis showed that LINC00460 expression was significantly upregulated in samples with advanced stage III/IV compared with that in stage I/II tumors (Table 1). Survival analysis showed that the high LINC00460 expression was significantly correlated with poor overall survival (OS) (*p* < 0.0001, Fig. 1e) and disease-free survival (DFS) (*p* = 0.0005, Fig. 1f). The Gene Expression Omnibus (GEO) data indicated that the high LINC00460 expression of CRC patients was correlated with poor OS and DFS (Fig. 1g–h). The GEO data also showed that LINC00460 expression was elevated in CRC in the advanced tumor stage (Fig. 1i). Finally, univariate analysis showed that LINC00460 was significantly associated with poor OS (hazard ratio [HR], 2.526; 95% confidence interval [CI], 1.696–3.761; *p* < 0.001) and DFS (HR, 3.437; 95% CI, 1.323–8.927; *p* = 0.001) (Additional file 4: Table S1). Multivariate analysis demonstrated that LINC00460 expression was also an independent prognostic marker for OS (HR, 2.299; 95% CI, 1.539–3.435; p < 0.001) and DFS (HR, 2.751; 95% CI, 1.052–7.198; *p =* 0.009) (Additional file 5: Table S2).

**Table 1:** Relationship between LINC00460 expression and clinicopathological features of CRC patients

Variables	All patients	LINC00460 expression		*P*-value^*^
		Low (%)	High (%)	
All cases	498	174 (35)	324 (65)	
Age				0.011
≤ 60 years	194	81 (42)	113 (58)	
>60 years	304	93 (31)	211 (69)	
Gender				0.700
Males	289	103 (36)	186 (64)	
Females	209	71 (34)	138 (66)	
TNM stage				0.005
I/II	275	109 (40)	166 (60)	
III/IV	223	65 (29)	158 (71)	
Lymph node metastasis				0.044
N0	302	116 (38)	186 (62)	
N1/N2/N3	196	58 (30)	138 (70)	
Metastasis				0.034
M0	461	167 (36)	294 (64)	
M1	37	7 (19)	30 (81)	
Tumor diameter				0.044
≤ 4.5 cm	251	77 (31)	174 (69)	
>4.5 cm	247	97 (39)	150 (61)	
Differentiation				0.959
Poor	427	149 (35)	278 (65)	
Moderate/High	71	25 (35)	46 (65)	
Depth of invasion				0.104
T1/T2	103	43 (42)	60 (58)	
T3/T4	395	131 (33)	264 (67)	

*P*-value^*^ measured by Pearson’s Chi-Squared test

### LINC00460 promotes CRC cell proliferation

**Fig. 2:**
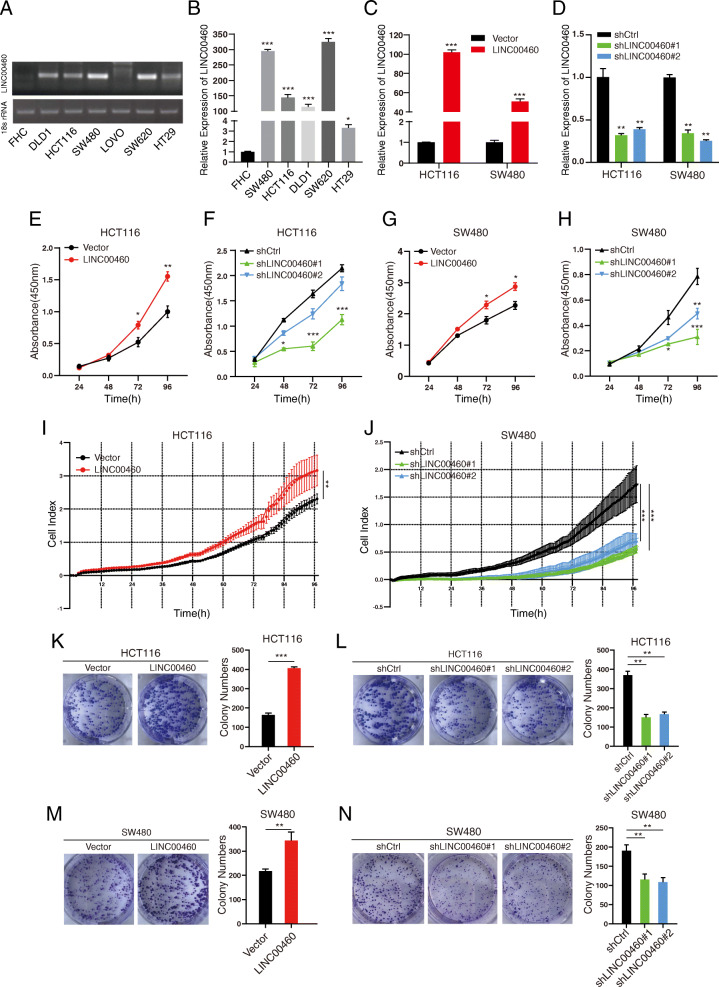
LINC00460 promotes CRC cell proliferation. **a**, **b** Detection of LINC00460 expression level by qRT-PCR and gel electrophoresis in colorectal cancer cell lines (DLD1, HCT116, SW480, LOVO, SW620, HT29) and compared with the human normal colorectal epithelial cell line (FHC). (*p < 0.05, ***p < 0.001) (**c**, **d**) Detection of LINC00460 level by qRT-PCR in LINC00460 KD and OE HCT116/SW480 stable cell lines. LINC00460 KD used the two of shRNAs (#1 and #2) of LINC00460 (**p < 0.01, ***p < 0.001). **e**, **f**, **g**, **h** Effect of LINC00460 KD or OE on HCT116 and SW480 cells proliferation as assessed by Cell Counting Kit-8 (CCK8) assays (*p < 0.05, **p < 0.01, ***p < 0.001). **i**, **j** Effect of LINC00460 KD or OE on HCT116 and SW480 cells proliferation as assessed by Real-Time Cell Analyzer (RTCA, XCELLigence) assays (**p < 0.01, ***p < 0.001). **k**, **l**, **m**, **o** Colony formation assays for LINC00460 KD or OE in HCT116/SW480 cell lines. The number of colonies with > 50 cells was counted after 2 weeks incubation (**p < 0.01, ***p < 0.001)

The endogenous expression of LINC00460 in colorectal cell lines, including DLD1, HCT116, SW480, LOVO, SW620, HT29, and the human normal colorectal epithelial cell FHC were measured by RT-PCR and gel electrophoresis. The results showed that CRC cells expressed higher LINC00460 compared with the normal colonic cell (Fig. 2a–b). Subsequently, stable knockdown or overexpression cell lines were established using shRNAs or LINC00460 overexpression lentivirus to investigate the biological roles of LINC00460 in CRC (Fig. 2c-d). Then, Cell Counting Kit-8 (CCK8) assays and a Real-Time Cell Analyzer (RTCA, xCELLigence) were used to examine the effect of LINC00460 on cell proliferation. CCK8 assay showed that LINC00460 overexpression increased HCT116 and SW480 cell proliferation (Fig. 2e–g). Conversely, HCT116 and SW480 cells depleted of LINC00460 displayed reduced rates of cell growth compared with the corresponding controls (Fig. 2f–h). The RTCA assays also LINC00460 positively regulated the proliferation of CRC cells (Fig. 2i-j, Additional file 6: Figure S2A-B). Colony formation assays suggested that LINC00460 promotes tumor cell growth in CRC cells (Fig. 2k-n). These results have revealed that LINC00460 is an important regulator of CRC cell proliferation.

### LINC00460 promotes CRC cell migration and invasion

**Fig. 3:**
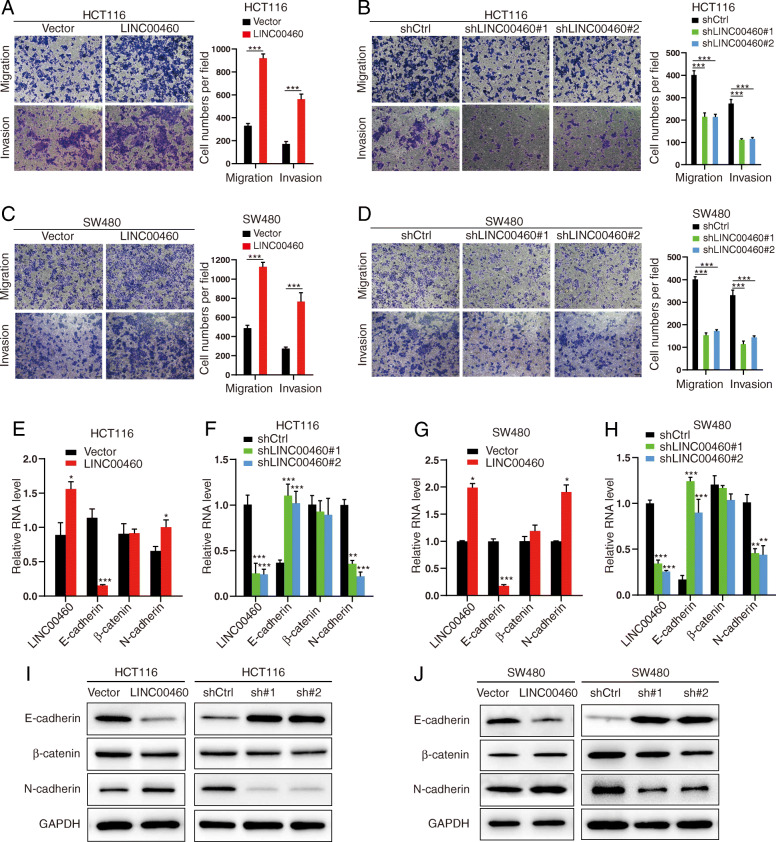
LINC00460 promotes CRC cell migration, invasion and supports the proper function of the EMT transcription program and the location of LINC00460 in CRC cells. **a**, **b**, **c**, **d** The ability of migration and invasion in HCT116 and SW480 cells ± LINC00460 KD/OE (***p < 0.001). **e**, **f**, **g**, **h** Relative mRNA expression levels of LINC00460 and EMT markers in HCT116 and SW480 cells ± LINC00460 KD/OE. The relative mRNA expression levels were normalized to GAPDH (*p < 0.05, **p < 0.01, ***p < 0.001). **i**, **j** Western blots of EMT markers in HCT116 and SW620 cells ± LINC00460 KD/OE. GAPDH was used as a loading control

To estimate the in the migration and invasion abilities of LINC00460 in CRC cells, we used transwell and wound healing assays to analyze the effects of LINC00460 on CRC cell migration and invasion. The results revealed that LINC00460 overexpression increased migration and invasion abilities, whereas LINC00460 knockdown decreased the abilities in HCT116 and SW480 cells (Fig. 3a-d). Wound healing assays revealed that LINC00460 positively regulated the wound healing speed in HCT116 and SW480 cells (Additional file 7: Figure S3A-D).

Given that Epithelial Mesenchymal Transition (EMT) is a well-known driving force for cell migration and invasion, we investigated the effects of LINC00460 on EMT. We observed that LINC00460 overexpression reduced the mRNA expression of E-cadherin and increased the mRNA expression of N-cadherin, whereas LINC00460 knockdown increased the mRNA level of E-cadherin and decreased N-cadherin mRNA expression (Fig. 3e-h). Consistent with mRNA changes, the protein levels of E-cadherin and N-cadherin were also altered via LINC00460 overexpression or knockdown in CRC cells (Fig. 3i-j). These results suggest that LINC00460 is essential for CRC cell migration and invasion.

### LINC00460 interacts with IGF2BP2 and DHX9 protein

**Fig. 4:**
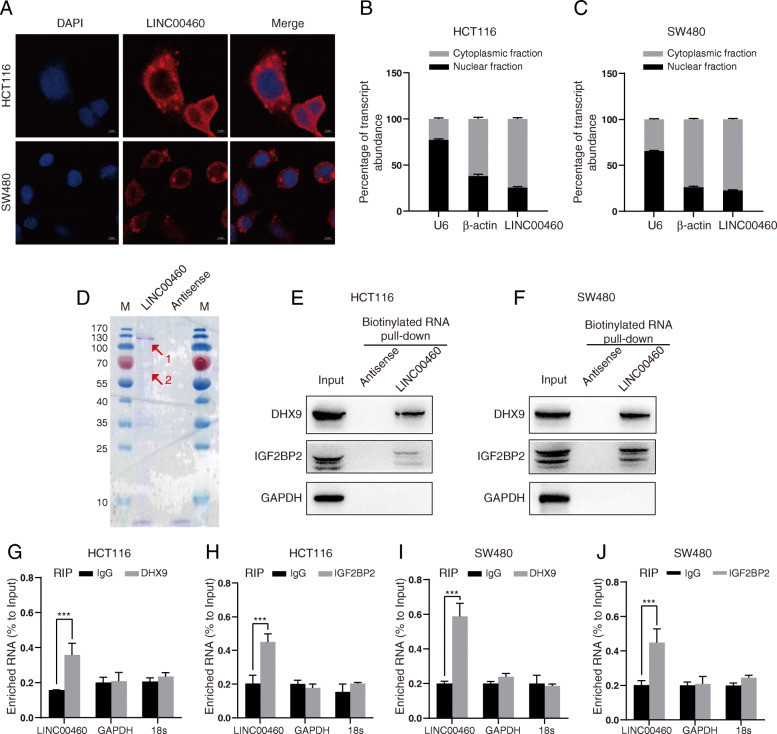
LINC00460 interacts with IGF2BP2 and DHX9 protein. **a** LINC00460 intracellular localization was visualized in CRC cells: HCT116, SW480, by FISH analysis. **b**, **c** Relative LINC00460 levels in HCT116 and SW480 cell cytoplasm or nucleus were detected by qRT-PCR. U6 was used as nuclear control and β-actin was used as cytoplasm control. **d** Coomassie Blue staining of biotinylated LINC00460-associated proteins is shown (1: DHX9; 2: IGF2BP2, The proteins was identified by mass spectrometry). **e**, **f** The interactions between LINC00460,DHX9 and IGF2BP2 identified by RNA-Pull down assays and Western blots in HCT116 and SW480 cells. Biotin labeled LINC00460 and its antisense RNA were in vitro transcribed by using T7 RNA polymerase. GAPDH was used as a negative control. **g**, **h**, **i**, **j** Results from RIP and subsequent qRT-PCR assays. RIP assays were performed to detect the interactions of LINC00460 and IFG2BP2/DHX9 in HCT116 and SW480 cells. Relative quantification of LINC00460, GAPDH, and 18S rRNA in RNA-protein complexes immunoprecipitated with IgG or DHX9, IGF2BP2 antibodies from whole cell extracts. Relative expressions were normalized by the input RNA, GAPDH and 18 s rRNA were used as negative control binding RNAs. Error bars indicate mean ± SD, n = 3 for technical replicates. ***p < 0.001

To investigate the regulatory mechanism of LINC00460 in CRC, the cellular localization of LINC00460 in CRC cells was studied using RNA fluorescence in situ hybridization (FISH) and subcellular fractionation assays. The results showed that LINC00460 was mainly localized in the cytoplasm of colon cancer cells (Fig. 4a-c). In additional, RNA ISH experiments showed that LINC00460 was predominantly localized cytoplasm in the CRC tissue samples (Additional file 8: Figure S4).

Evidence has proven that lncRNAs may interact with binding factors and modulate their downstream target genes. Therefore, we performed RNA pull-down assay to identify the binding proteins of LINC00460. The retrieved proteins were subjected to SDS-PAGE electrophoresis analysis, and the differential bands were selected for a Mass Spectrometry. Among the proteins identified by mass spectrum analysis, we identified two proteins, namely, IGF2BP2 and DHX9, that directly interacted with LINC00460 (Fig. 4d). RNA pull-down and Western blot assays further verified that LINC00460 could interact with IGF2BP2 and DHX9 in HCT116 and SW480 cells (Fig. 4e-f). For the specificity of interactions between LINC00460 and IGF2BP2/DHX9, we performed RNA Immunoprecipitation (RIP) assays to validation by using IGF2BP2/DHX9 antibody in HCT116 and SW480 cells (Fig. 4g-j). Interestingly, IGF2BP2 and DHX9 have been shown to interact with each other [28].

### LINC00460 mediates the regulation of HMGA1 mRNA stability

**Fig. 5:**
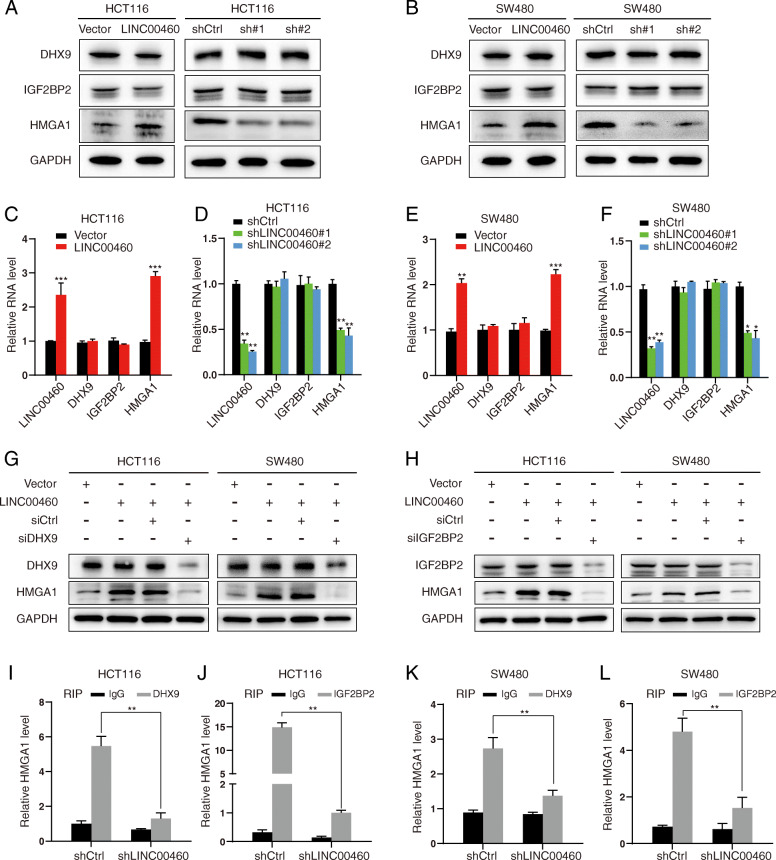
LINC00460 mediated regulation of HMGA1 mRNA stability. **a**, **b** Effect of LINC00460 KD or OE on the expression of IGF2BP2, DHX9 and HMGA1 protein level in HCT116 and SW480 cells, as assessed by Western blot. **c**, **d**, **e**, **f** Relative mRNA expression levels of LINC00460, IGF2BP2, DHX9 and HMGA1 in HCT116 and SW480 cells ± LINC00460 KD/OE (*p < 0.05, **p < 0.01, ***p < 0.001). **g**, **h** Western blot assay was performed to measure DHX9, IFG2BP2 and HMGA1 protein level after transfection of DHX9/IGF2BP2 siRNA, control siRNA in LINC00460 overexpressed CRC cells. **i**, **j**, **k**, **l** RIP assay shows interaction declined between HMGA1 and DHX9/IGF2BP2 after knockdown of LINC00460 in CRC cells

One of the prominent mechanisms of lncRNAs is their capability to interact with other cellular factors, including proteins, DNA, and other RNA molecules [29]. Marco Mineo et al.(2016) have demonstrated that lncRNA HIF1A-AS2 binding proteins IGF2BP2 and DHX9 are engaged in downstream mRNA stabilization [21]. Meanwhile, among the IGF2BP2 target genes, HMGA1 and FOSL1 are also regulated by DHX9 [30]. Interestingly, LINC00460 showed no interaction with protein chromatin-remodeling complexes nor transcriptional machinery in contrast to previous reports on other lncRNAs. We propose a hypothesis that LINC00460 interacts with IGF2BP2 and DHX9 and affects the mRNA stabilization of downstream targets. Western blot analysis was used to validate whether the interaction of LINC00460 and its protein partners resulted in functional consequences for their downstream target. The results showed LINC00460 knockdown or overexpression exhibited no influence on IGF2BP2 or DHX9 protein expression, and it positively regulated the HMGA1 protein level (Fig. 5a-b), as well as the mRNA (Fig. 5c-f).

We showed that knockdown of DHX9 or IGF2BP2 significantly inhibited the LINC00460-induced HMGA1 upregulation in HCT116 and SW480 cells (Fig. 5g-h). RIP assays showed that knockdown of LINC00460 resulted in significantly diminished interactions between DHX9 or IGF2BP2 and HMGA1 (Fig. 5i-l). All the data suggest that LINC00460, IGF2BP2, and DHX9 may cooperate to regulate HMGA1 post-transcriptional mRNA stabilization and enhance HMGA1 expression.

### LINC00460 promotes CRC growth and metastasis by regulating HMGA1

**Fig. 6:**
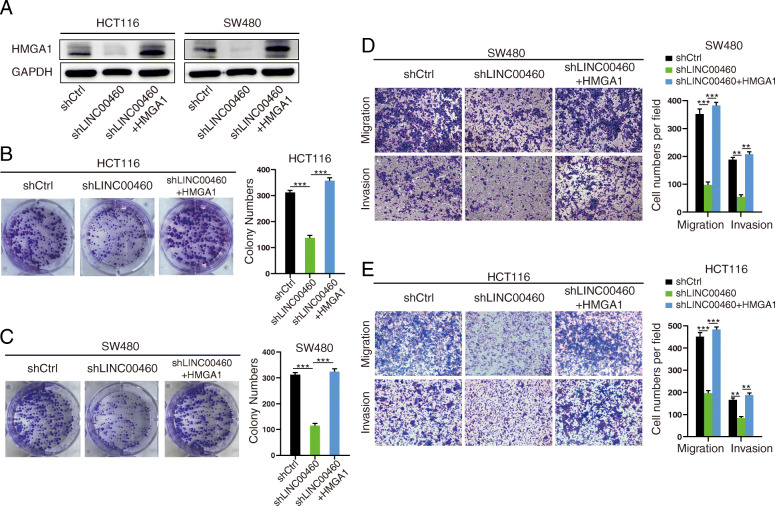
LINC00460 governs CRC growth and metastasis by stabilizing HMGA1 mRNA in vitro. **a** The HMGA1 protein level analyzed by Western blot after re-express HMGA1 in LINC00460 KD CRC cells. **b**, **c** Colony formation assays for effects of HMGA1 re-expression on proliferation of HCT116 and SW480 cells with LINC00460 KD (**p < 0.01, ***p < 0.001). **d**, **e** Effects of HMGA1 re-expression on migration and invasion of HCT116 and SW480 cells with LINC00460 KD (**p < 0.01, ***p < 0.001)

We hypothesized that HMGA1 mediated the biological function of LINC00460 in CRC. To test this hypothesis, we re-expressed HMGA1 in LINC00460 knockdown CRC cells (Fig. 6a). The re-expression of HMGA1 dramatically rescued the proliferation abilities of HCT116 and SW480 cells (Fig. 6b-c). Furthermore, HMGA1 re-expression in LINC00460 knockdown cells remarkably increased the migration and invasion abilities of the studied cell lines (Fig. 6d-e).

**Fig. 7:**
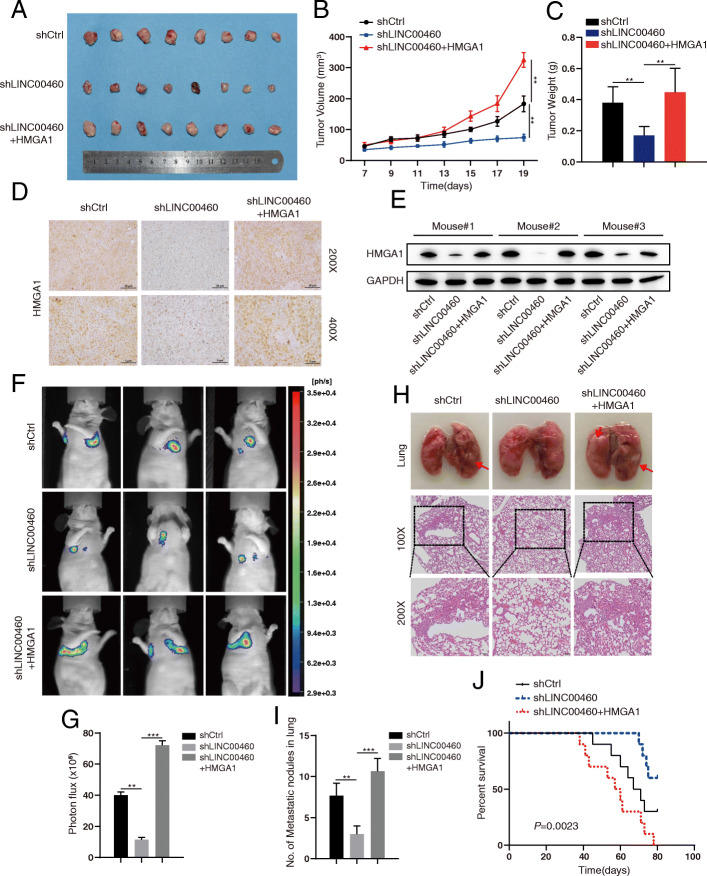
LINC00460 governs CRC growth and metastasis by stabilizing HMGA1 mRNA in vivo. **a**, **b**, **c** Effect of LINC00460 KD and LINC00460 KD with overexpression HMGA1 in HCT116 cells on the xenograft model was assessed by evaluating tumor volume and tumor weight. 5 × 10^6^ HCT116 LINC00460 Con/KD cells and LINC00460 KD with overexpression HMGA1 and Matrigel (Corning; 1:1 ratio) were subcutaneously injected into the abdominal flanks of each mice, respectively (**p < 0.01). **d** The tumor sections were performed immunochemistry staining by antibody against HMGA1, representative images were shown. **e** Western blot analysis of HMGA1 expression in tumor xenografts formed. GAPDH was used as a loading control. **f**, **g** Representative bioluminescence images and statistical analysis of lung metastases in mice via tail vein injection of indicated cells (**p < 0.01, ***p < 0.001). **h** HCT116 LINC00460 Con/KD cells and LINC00460 KD with overexpression HMGA1 cells were injected via the lateral tail veins. Representative lung images (up) at week 6, corresponding hematoxylin eosin stained lung sections are shown (low). Arrows, lung metastasis foci. i Statistical analysis of lung metastasis foci (**p < 0.01, ***p < 0.001). **j** Kaplan-Meier survival curves depicting overall survival of the mice with injection of HCT116 LINC00460 Con/KD cells and LINC00460 KD with overexpression HMGA1 (**p < 0.01, ***p < 0.001)

We performed xenograft experiments and assessed the effects of LINC00460 and HMGA1 in LINC00460 knockdown CRC cells by using nude mice to demonstrate their effects on tumor growth in vivo. Consistent with the in vitro assays, the tumor growth of LINC00460 knockdown group was notably slower than that of the control group (Fig. 7a), occurring together with the significantly decreased tumor volume and weights (Fig. 7b and c respectively). HMGA1 re-expression dramatically rescued tumor volume and weights affected by the LINC00460 knockdown (Fig. 7a-c). HMGA1 expression was evaluated via IHC staining, Western blots, and RT-PCR of tissue sections from nude mice (Fig. 7d-e, Additional file 9: Figure S5).

Given that migration and invasion are essential in cancer metastasis, we investigated the role of LINC00460 in CRC metastasis in vivo. HCT116-Luc-shCtrl, HCT116-Luc-shLINC00460, and HCT116-Luc-shLINC00460 + HMGA1 cells were injected into the tail veins of nude mice respectively. HCT116-Luc-shCtrl and HCT116-Luc-shLINC00460 + HMGA1 CRC cells effectively metastasized to the lung region of nude mice in 6 weeks (Fig. 7f), whereas HCT116-Luc-shLINC00460 cells showed no such result, as shown by bioluminescence imaging (Fig. 7f-g). LINC00460 knockdown cells formed fewer metastatic foci in lungs than the control group (Fig. 7h), whereas the re-expression of HMGA1 in cells formed more metastatic foci in the lungs than those in the LINC00460 knockdown cells (Fig. 7h-i). Furthermore, we calculated the mice’s survival status. LINC00460 knockdown increased the survival rates, whereas HMGA1 re-expression caused the opposite result (Fig. 7j). Therefore, LINC00460 governs CRC growth and metastasis by regulating HMGA1 mRNA stabilization in vivo.

### LINC00460 regulates HMGA1 expression depending on METTL3-mediating m^6^A modification of HMGA1 mRNA

**Fig. 8:**
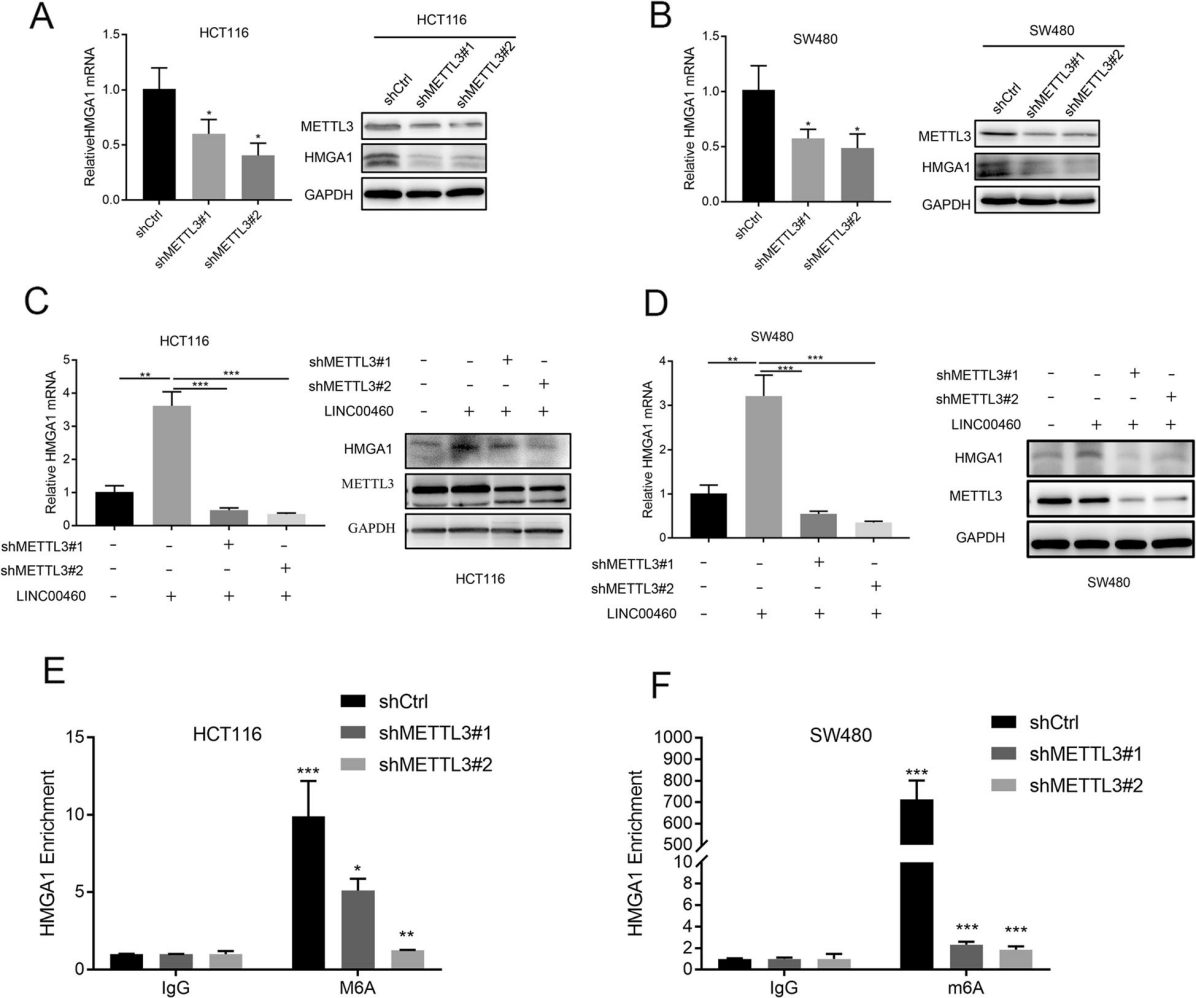
METTL3-Mediated modification of HMGA1 stabilized HMGA1 expression in CRC. **a-b** qRT-PCR and western blot assays showed the mRNA and protein expression, respectively, of HMGA1 in HCT116 and SW480 cells with METTL3 knockdown. Results was normalized with GAPDH (*p < 0.05). **c-d** qRT-PCR and western blot assays showed the mRNA and protein expression of HMGA1 in LINC00460 overexpression HCT116 and SW480 cells with METTL3 knockdown. Results was normalized with GAPDH (**p < 0.01, ***p < 0.001). **e-f** m^6^A immunoprecipitation and qRT-PCR assays showed the relative expression HMGA1 mRNA with methylation. Relative HMGA1 fold changes were normalized with the input, IgG was used as a negative control (*p < 0.05, **p < 0.01, ***p < 0.001)

Considering LINC00460 interacting with IGF2BP2 and IGF2BP2 has been suggested as a m^6^A reader governing mRNA stability. We assumed that LINC00460 may regulate HMGA1 mRNA stability depending on m^6^A modification. To confirm this hypothesis, we checked the HMGA1 mRNA m^6^A modification status using the MethylTranscriptome DataBase Version 2.0 prediction tool (MeT-DB V2.0) [31], Results showed that the 3’UTR region of HMGA1 mRNA have a significant m6A modification signaling, and the 3’UTR region of HMGA1 also contains several METTL3 binding sequences RRACH (R = G/A and H = A/C/U) (Additional file 10: Figure S6A-B). We wondered whether m^6^A modification regulates the mRNA stability of HMGA1, silencing the expression of METTL3, an important m6A methyltransferase, decreased HMGA1 expression (Fig. 8a-b). Furthermore, we showed that silencing METTL3 in LINC00460 overexpression cells could significantly reduce HMGA1 expression (Fig. 8c-d). Finally, MeRIP was used to test the m^6^A modification status of HMGA1 mRNA; results showed that silencing METTL3 could significantly decrease the m6A modification status of HMGA1 mRNA (Fig. 8e-f). These results suggested that LINC00460 may regulate HMGA1 expression in a m6A modification dependent manner.

## Discussion

Accumulating evidence has indicated that lncRNAs play vital roles in human cancer. However, limited information is known about their pattern of action and potential role in the regulation of cancer-related processes. To our knowledge, LINC00460 has been reported to participate in many kinds of malignancies and promotes cancer progressions [15–20]. Although, some kinds of molecular mechanism of LINC00460 in cancer progression have been reported, the potential mechanism in regulating CRC progression needs further explored. In the present work, we revealed the potential clinical diagnosis or prognosis value and uncovered a novel molecular mechanism of LINC00460 in regulating CRC metastasis.

In previous studies, LINC00460 have been proved overexpressed in CRC by using TCGA data and conforming by a small CRC cohort detected by qRT-PCR. In this study, RNAscope probes were used to detect the expression in a CRC cohort containing 498 CRC tissues and corresponding non-tumor adjacent tissues. Our findings showed that LINC00460 expression increased in human CRC, and high LINC00460 expression was correlated with clinicopathological parameters, such as TNM stage, lymph node metastasis, metastasis, and tumor size. Furthermore, a high LINC00460 expression indicated poor five-year OS and DFS via tissue microarrays. Univariate and Multivariate Cox regression analyses showed that high LINC00460 expression is an independent adverse prognostic factor for CRC patients. Our findings provided reliable evidence that LINC00460 could act as a potential clinical prognostic predictor for patients with CRC.

To further validate the biological function of LINC00460 in CRC progression, we investigated the malignant features of LINC00460 in a series of in vitro and in vivo experiments. CRC cell lines HCT116 and SW480 were studied using LINC00460 knockdown or the overexpress lentivirus infection. Consistent with these previous data, our results showed that LINC00460 KD and OE respectively reduced or increased tumor cell growth, migration, and invasion abilities. LINC00460 was shown to support the proper functioning of the EMT transcription program, downregulate epithelial markers (such as E-cadherin), and upregulate mesenchymal markers (such as N-cadherin). Our data also showed that LINC00460 downregulation suppressed tumor growth and lung metastasis in a xenograft mouse model. These results consistently emphasized the importance of LINC00460 in CRC progression.

Several studies have suggested LINC00460 was mainly located in cytoplasm [17–19]. In the present study, we also showed that LINC00460 is mainly distributed in the cell cytoplasm. Cytoplasmic lncRNAs may play a role as a regulator of transcription and/or protein stabilization and modification [32, 33]. Previous studies showed LINC00460 could bind to miR-433-3p to promote CRC EMT [19], and also could bind to miR-149-5p and miR150-5p to promotes Oxaliplatin resistance in CRC [20]. In this study, we discovered that LINC00460 could interact with Insulin-like growth factor 2 mRNA-binding protein 2 (IGF2BP2) and ATP-dependent RNA helicase A (DHX9). IGF2BP2 is a member of the RNA binding protein family (IMP1, IMP2 and IMP3), which is involved in RNA localization, translation, and stability [34]. DHX9, also known as RNA helicase A, belongs to the DExH-box family of helicase proteins and plays critical roles in multiple levels of gene regulation, including transcription [35, 36], translation [37, 38], RNA processing and transport [39, 40], and maintenance of genomic stability [41, 42]. Chatel-Chaix et al. (2013) have proven that IGF2BP2 and DHX9 interact with each other [21]. Marco Mineo et al. (2016) further reported that lncRNA HIF1A-AS2 could interact with IGF2BP2 and DHX9 to form a complex and maintain their downstream target HMGA1 mRNA stability to promote glioblastoma. We showed LINC00460 directly interacts with IGF2BP2 and DHX9 to regulate HMGA1 mRNA stability. Considering IGF2BP2 functions as a m^6^A reader and the 3’UTR of HMGA1 mRNA have a strong m^6^A modification signal. We then showed that silencing METTL3 could affect LINC00460 induced HMGA1 expression, suggesting LINC00460/DHX9/IGF2BP2 complex may stabilize the mRNA of HMGA1 through recognizing m^6^A modification.

High mobility group proteins are a family of non-histone chromatin-bound proteins without any transcriptional activity; however, they modulate transcription by altering the architecture of chromatin [43, 44] and organize the assembly of several transcription factors to enhanceosomes [45–51]. In addition to HMGA1’s function during development [52], high mobility group proteins are abnormally expressed and localized in virtually every cancer [53], and their expression levels correlate with the degree of malignancy. In a series of rescue experiments, we demonstrated that HMGA1 re-expression dramatically rescued the proliferation, migration, and invasion of CRC tumors and cells decreased by LINC00460 silencing in vitro and in vivo experimental models. The malignant effects of HMGA1 on CRC growth and metastasis were validated.

## Conclusion

In summary, we have exhibited a set of comprehensive data suggesting the clinical and functional significance of LINC00460 in CRC proliferation and metastasis. We uncovered a novel molecular mechanism of LINC00460 in regulating CRC progression. We conclude LINC00460/DHX9/IGF2BP2 complex may regulate HMGA1 expression through recognizing the m6A modification sites of HMGA1 to enhance its mRNA stability and promote CRC metastasis. LINC00460 may serve as potential biomarker and target in the prognosis and treatment of CRC.

## Supplementary Information

**Additional file 1: Table S1.** Primers used for qRT-PCR.

**Additional file 2: Dataset 1.** Differentially expressed genes in the microarray.

**Additional file 3: Figure S1.** PCR detection of LINC00460 in CRC tissues and corresponding adjacent normal colon tissues using gel electrophoresis

**Additional file 4: Table S2.** Univariate Cox regression analysis of LINC00460 expression and clinicopathologic variables predicting the survival of CRC patients

**Additional file 5: Table S3.** Multvariate Cox regression analysis of LINC00460 expression and clinicopathologic variables predicting the survival of CRC patients

**Additional file 6: Figure S2.** RTCA assays showed LINC00460 positively regulated CRC cells proliferation. (A, B) Effect of LINC00460 KD or OE on HCT116 and SW480 cells proliferation as assessed by Real-Time Cell Analyzer (RTCA, XCELLigence) assays (*p < 0.05, **p < 0.01, ***p < 0.001).

**Additional file 7: Figure S3.** Wound-healing assays showed LINC00460 positively regulated CRC cell mobility. (A, B, C, D) The ability of motility in HCT116 and SW480 cells ± LINC00460 KD/OE tested by wound-healing assay. Statistical analysis was performed using unpaired t-tests. All statistical tests were two-sided. *p < 0.05, **p < 0.01, ***p < 0.001.

**Additional file 8: Figure S4.** LINC00460 intracellular localization in CRC tissues.

**Additional file 9: Figure S5.** Relative mRNA expression levels of HMGA1 expression in tumor xenografts.

**Additional file 10: Figure S6.** Prediction of HMGA1 mRNA m6A modification status. (A) HMGA1 mRNA m^6^A modification status in the MethylTranscriptome DataBase v2.0. (B) Potential METTL3 modified regions in the 3’UTR of HMGA1 mRNA.

## Data Availability

The datasets used and/or analyzed during the current study are available from the corresponding author on reasonable request.

## Abbreviations

lncRNAs: Long noncoding RNAs
CRC: Colorectal carcinoma
LINC00460: Long intergenic noncoding RNA 460;
EMT: Epithelial-mesenchymal transition
HMGA1: High-mobility group AT-hook 1
IGF2BP2: Insulin like growth factor 2 mRNA binding protein 2
DHX9: ATP-dependent RNA helicase A
3′UTR: 3′ untranslated region
m6A: The N6-methyladenosine
METTL3: Methyltransferase-like 3
CCAT2: Colon Cancer Associated Transcript 2
MALAT1: Metastasis-associated lung adenocarcinama transcript 1
NSCLC: Non-small cell lung cancer
HIF1A-AS2: Hypoxia-inducible factor 1 alpha (HIF1A)-antisense RNA 2
TMAs: Tissue microarrays
siRNA: Small interfering RNAs
shRNA: Short hairpin RNA
RT-PCR: Real-time PCR CCK-8: Cell Counting Kit-8 ISH: In situ hybridization
IHC: Immunohistochemical
FISH: RNA fluorescence in situ hybridization
RTCA: Real-time Cellular Analysis
RIP: RNA immunoprecipitation
MS: Mass spectrometry
MeRIP: Methylated RNA immunoprecipitation
RT-PCR: Reverse transcription-PCR
NCTs: Normal colorectal tissues
DFS: Disease-free survival
GEO: The Gene Expression Omnibus
OS: Overall survival
HR: Hazard ratio
CI: Confidence interval
KD: Knockdown
OE: Overexpression

## References

[1] Lian Y, Cai Z, Gong H, Xue S, Wu D, Wang K (2016). HOTTIP: a critical oncogenic long non-coding RNA in human cancers. Mol BioSyst.

[2] Deniz E, Erman B (2017). Long noncoding RNA (lincRNA), a new paradigm in gene expression control. Funct Integr Genomics.

[3] Fatica A, Bozzoni I (2014). Long non-coding RNAs: new players in cell differentiation and development. Nat Rev Genet.

[4] Bolha L, Ravnik-Glavac M, Glavac D (2017). Long noncoding RNAs as biomarkers in Cancer. Dis Markers.

[5] Huarte M (2015). The emerging role of lncRNAs in cancer. Nat Med.

[6] Ling H, Spizzo R, Atlasi Y, Nicoloso M, Shimizu M, Redis RS (2013). CCAT2, a novel noncoding RNA mapping to 8q24, underlies metastatic progression and chromosomal instability in colon cancer. Genome Res.

[7] Ji P, Diederichs S, Wang W, Boing S, Metzger R, Schneider PM (2003). MALAT-1, a novel noncoding RNA, and thymosin beta4 predict metastasis and survival in early-stage non-small cell lung cancer. Oncogene..

[8] Kogo R, Shimamura T, Mimori K, Kawahara K, Imoto S, Sudo T (2011). Long noncoding RNA HOTAIR regulates polycomb-dependent chromatin modification and is associated with poor prognosis in colorectal cancers. Cancer Res.

[9] Xiang J-F, Yin Q-F, Chen T, Zhang Y, Zhang X-O, Wu Z (2014). Human colorectal cancer-specific CCAT1-L lncRNA regulates long-range chromatin interactions at the MYC locus. Cell Res.

[10] Chen W, Zheng R, Baade PD, Zhang S, Zeng H, Bray F (2016). Cancer statistics in China, 2015. CA Cancer J Clin.

[11] Seymour MT, Thompson LC, Wasan HS, Middleton G, Brewster AE, Shepherd SF (2011). Chemotherapy options in elderly and frail patients with metastatic colorectal cancer (MRC FOCUS2): an open-label, randomised factorial trial. Lancet.

[12] Walsh JM, Terdiman JP (2003). Colorectal cancer screening: scientific review. JAMA..

[13] Andre N, Schmiegel W (2005). Chemoradiotherapy for colorectal cancer. Gut..

[14] Landreau P, Drouillard A, Launoy G, Ortega-Deballon P, Jooste V, Lepage C (2015). Incidence and survival in late liver metastases of colorectal cancer. J Gastroenterol Hepatol.

[15] Yang J, Lian Y, Yang R, Lian Y, Wu J, Liu J (2020). Upregulation of lncRNA LINC00460 facilitates GC progression through epigenetically silencing CCNG2 by EZH2/LSD1 and indicates poor outcomes. Mol Ther Nucleic Acids..

[16] Li K, Sun D, Gou Q, Ke X, Gong Y, Zuo Y (2018). Long non-coding RNA linc00460 promotes epithelial-mesenchymal transition and cell migration in lung cancer cells. Cancer Lett.

[17] Jiang Y, Cao W, Wu K, Qin X, Wang X, Li Y (2019). LncRNA LINC00460 promotes EMT in head and neck squamous cell carcinoma by facilitating peroxiredoxin-1 into the nucleus. J Exp Clin Cancer Res.

[18] Tu J, Zhao Z, Xu M, Chen M, Weng Q, Ji J (2019). LINC00460 promotes hepatocellular carcinoma development through sponging miR-485-5p to up-regulate PAK1. Biomed Pharmacother.

[19] Hong W, Ying H, Lin F, Ding R, Wang W, Zhang M (2020). lncRNA LINC00460 silencing represses EMT in Colon Cancer through Downregulation of ANXA2 via Upregulating miR-433-3p. Mol Ther Nucleic Acids.

[20] Meng X, Sun W, Yu J, Zhou Y, Gu Y, Han J (2020). LINC00460-miR-149-5p/miR-150-5p-mutant p53 feedback loop promotes Oxaliplatin resistance in colorectal Cancer. Mol Ther Nucleic Acids..

[21] Mineo M, Ricklefs F, Rooj AK, Lyons SM, Ivanov P, Ansari KI (2016). The long non-coding RNA HIF1A-AS2 facilitates the maintenance of Mesenchymal Glioblastoma stem-like cells in hypoxic niches. Cell Rep.

[22] Fedele M, Fusco A (2010). HMGA and cancer. Biochim Biophys Acta.

[23] Dai N, Ji F, Wright J, Minichiello L, Sadreyev R, Avruch J. IGF2 mRNA binding protein-2 is a tumor promoter that drives cancer proliferation through its client mRNAs IGF2 and HMGA1. eLife. 2017;6:27155. 10.7554/eLife.27155 PMC5576481 28753127

[24] Huang H, Weng H, Sun W, Qin X, Shi H, Wu H (2018). Recognition of RNA N (6)-methyladenosine by IGF2BP proteins enhances mRNA stability and translation. Nat Cell Biol.

[25] Vu LP, Pickering BF, Cheng Y, Zaccara S, Nguyen D, Minuesa G (2017). The N(6)-methyladenosine (m(6)a)-forming enzyme METTL3 controls myeloid differentiation of normal hematopoietic and leukemia cells. Nat Med.

[26] Wang X, Lu Z, Gomez A, Hon GC, Yue Y, Han D (2014). N6-methyladenosine-dependent regulation of messenger RNA stability. Nature..

[27] Hou PF, Jiang T, Chen F, Shi PC, Li HQ, Bai J (2018). KIF4A facilitates cell proliferation via induction of p21-mediated cell cycle progression and promotes metastasis in colorectal cancer. Cell Death Dis.

[28] Chatel-Chaix L, Germain MA, Motorina A, Bonneil E, Thibault P, Baril M (2013). A host YB-1 ribonucleoprotein complex is hijacked by hepatitis C virus for the control of NS3-dependent particle production. J Virol.

[29] Minajigi A, Froberg J, Wei C, Sunwoo H, Kesner B, Colognori D, et al. Chromosomes. A comprehensive Xist interactome reveals cohesin repulsion and an RNA-directed chromosome conformation. Science. 2015;349(6245):aab2276. 10.1126/science.aab2276 PMC4845908 26089354

[30] Lee T, Di Paola D, Malina A, Mills JR, Kreps A, Grosse F (2014). Suppression of the DHX9 helicase induces premature senescence in human diploid fibroblasts in a p53-dependent manner. J Biol Chem.

[31] Liu H, Wang H, Wei Z, Zhang S, Hua G, Zhang SW (2018). MeT-DB V2.0: elucidating context-specific functions of N6-methyl-adenosine methyltranscriptome. Nucleic Acids Res.

[32] Wang P, Xue Y, Han Y, Lin L, Wu C, Xu S (2014). The STAT3-binding long noncoding RNA lnc-DC controls human dendritic cell differentiation. Science..

[33] Li Z, Zhang J, Liu X, Li S, Wang Q, Di C (2018). The LINC01138 drives malignancies via activating arginine methyltransferase 5 in hepatocellular carcinoma. Nat Commun.

[34] Christiansen J, Kolte AM, Hansen T, Nielsen FC (2009). IGF2 mRNA-binding protein 2: biological function and putative role in type 2 diabetes. J Mol Endocrinol.

[35] Nakajima T, Uchida C, Anderson SF, Lee CG, Hurwitz J, Parvin JD (1997). RNA helicase a mediates association of CBP with RNA polymerase II. Cell..

[36] Tetsuka T, Uranishi H, Sanda T, Asamitsu K, Yang JP, Wong-Staal F (2004). RNA helicase a interacts with nuclear factor kappaB p65 and functions as a transcriptional coactivator. Eur J Biochem.

[37] Hartman TR, Qian S, Bolinger C, Fernandez S, Schoenberg DR, Boris-Lawrie K (2006). RNA helicase a is necessary for translation of selected messenger RNAs. Nat Struct Mol Biol.

[38] Manojlovic Z, Stefanovic B (2012). A novel role of RNA helicase a in regulation of translation of type I collagen mRNAs. RNA..

[39] Bratt E, Ohman M (2003). Coordination of editing and splicing of glutamate receptor pre-mRNA. RNA..

[40] Tang H, Gaietta GM, Fischer WH, Ellisman MH, Wong-Staal F (1997). A cellular cofactor for the constitutive transport element of type D retrovirus. Science..

[41] Jain A, Bacolla A, Del Mundo IM, Zhao J, Wang G, Vasquez KM (2013). DHX9 helicase is involved in preventing genomic instability induced by alternatively structured DNA in human cells. Nucleic Acids Res.

[42] Jain A, Bacolla A, Chakraborty P, Grosse F, Vasquez KM (2010). Human DHX9 helicase unwinds triple-helical DNA structures. Biochemistry..

[43] Pennisi E (2013). Long noncoding RNAs may alter chromosome's 3D structure. Science..

[44] Reeves R (2015). High mobility group (HMG) proteins: modulators of chromatin structure and DNA repair in mammalian cells. DNA Repair (Amst).

[45] Apostolou E, Thanos D (2008). Virus infection induces NF-kappaB-dependent interchromosomal associations mediating monoallelic IFN-beta gene expression. Cell..

[46] Thanos D, Maniatis T (1995). Virus induction of human IFN beta gene expression requires the assembly of an enhanceosome. Cell..

[47] Ford E, Thanos D (2010). The transcriptional code of human IFN-beta gene expression. Biochim Biophys Acta.

[48] Falvo JV, Thanos D, Maniatis T (1995). Reversal of intrinsic DNA bends in the IFN beta gene enhancer by transcription factors and the architectural protein HMG I(Y). Cell..

[49] Thanos D, Maniatis T (1992). The high mobility group protein HMG I(Y) is required for NF-kappa B-dependent virus induction of the human IFN-beta gene. Cell..

[50] Du W, Maniatis T (1994). The high mobility group protein HMG I(Y) can stimulate or inhibit DNA binding of distinct transcription factor ATF-2 isoforms. Proc Natl Acad Sci U S A.

[51] Panne D, Maniatis T, Harrison SC (2007). An atomic model of the interferon-beta enhanceosome. Cell..

[52] Chiappetta G, Avantaggiato V, Visconti R, Fedele M, Battista S, Trapasso F (1996). High level expression of the HMGI (Y) gene during embryonic development. Oncogene..

[53] Hock R, Furusawa T, Ueda T, Bustin M (2007). HMG chromosomal proteins in development and disease. Trends Cell Biol.

