# Correction to: LINC00460/DHX9/IGF2BP2 complex promotes colorectal cancer proliferation and metastasis by mediating HMGA1 mRNA stability depending on m6A modification

**DOI:** 10.1186/s13046-021-02169-1

**Published:** 2021-11-16

**Authors:** Pingfu Hou, Sen Meng, Minle Li, Tian Lin, Sufang Chu, Zhongwei Li, Junnian Zheng, Yuming Gu, Jin Bai

**Affiliations:** 1 Cancer Institute, Xuzhou Medical University, 84 West Huaihai Road, Xuzhou, 221002 Jiangsu Province China; 2 Center of Clinical Oncology, Affiliated Hospital of Xuzhou Medical University, Xuzhou, China; 3 Department of Interventional Radiography, Affiliated Hospital of Xuzhou Medical University, 99 West Huaihai Road, Xuzhou, 221002 Jiangsu Province China

**Correction to: J Exp Clin Cancer Res 40, 52 (2021)**

**https://doi.org/10.1186/s13046-021-01857-2**

Following publication of the original article [1], the authors identified minor errors in Fig. 3, specifically:

- Fig. 3d: incorrect image was used for migration cells image of shLINC00460#2 (1^st^ row, 3^rd^ column)

The authors provided the Journal with the original data files. The corrected figure is provided here. The correction does not have any effect on the results or conclusions of the paper. The original article has been corrected.

**Fig. 3:**
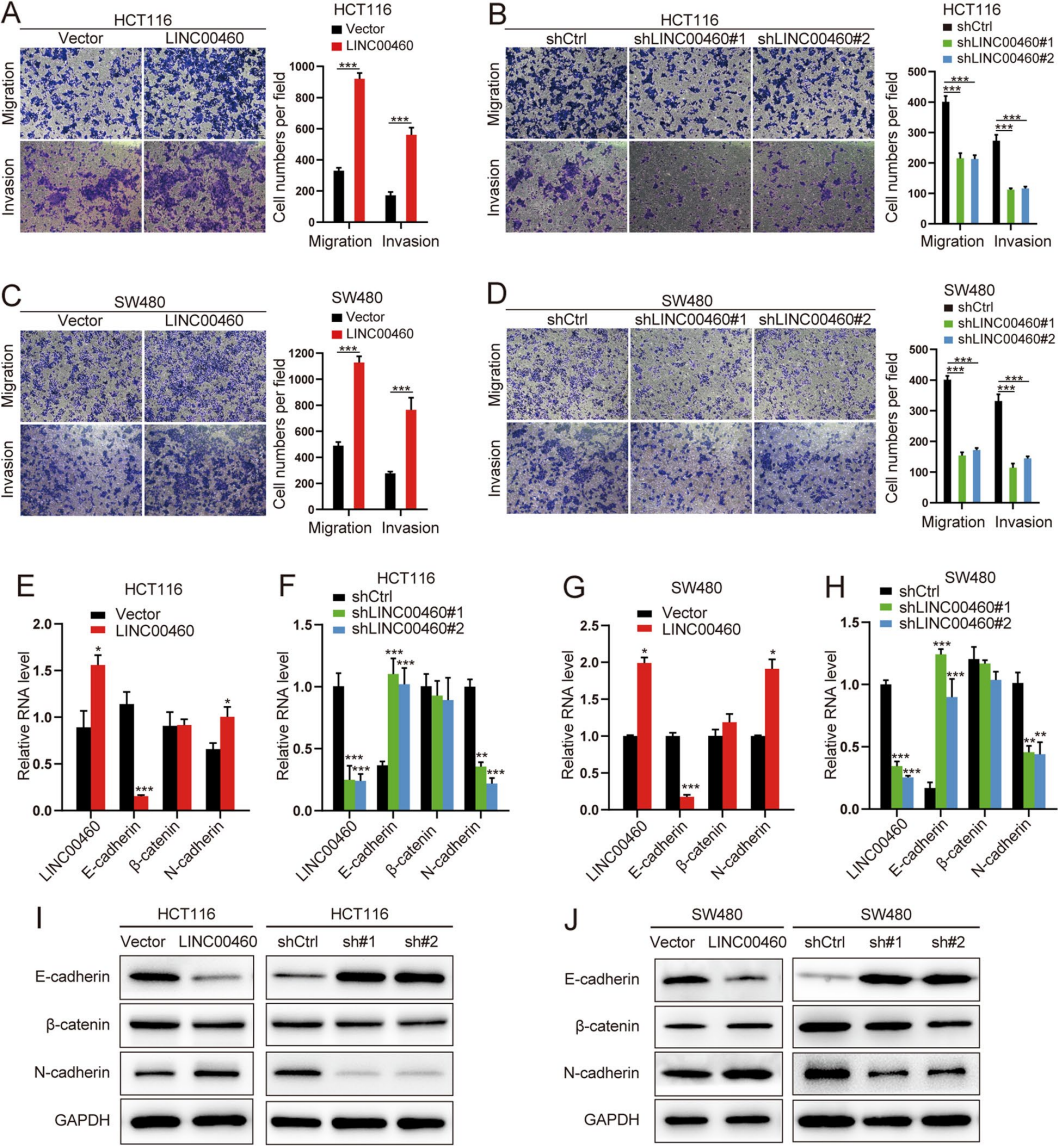
LINC00460 promotes CRC cell migration, invasion and supports the proper function of the EMT transcription program and the location of LINC00460 in CRC cells. **a, b, c, d** The ability of migration and invasion in HCT116 and SW480 cells ± LINC00460 KD/OE (***p < 0.001). **e, f, g, h** Relative mRNA expression levels of LINC00460 and EMT markers in HCT116 and SW480 cells ± LINC00460 KD/OE. The relative mRNA expression levels were normalized to GAPDH (*p < 0.05, **p < 0.01, ***p < 0.001). **i, j** Western blots of EMT markers in HCT116 and SW620 cells ± LINC00460 KD/OE. GAPDH was used as a loading control
