# Supplementary material for: LINC00460/DHX9/IGF2BP2 complex promotes colorectal cancer proliferation and metastasis by mediating HMGA1 mRNA stability depending on m6A modification
Source: J Exp Clin Cancer Res. 2021 Feb 1;40:52. doi: 10.1186/s13046-021-01857-2 (PMC7851923; doi:10.1186/s13046-021-01857-2)
Supplement: Supplementary file 1 — Additional file 1: Table S1. Primers used for qRT-PCR. [file 13046_2021_1857_MOESM1_ESM.docx]

**Table S1:** Primers used for q-RT-PCR

| LINC00460 Forward | ACGCAGTGGATGAGAACGAA |
| --- | --- |
| LINC00460 Reverse | GGGGTGACTTCAGAATGCGT |
| IGF2BP2 Forward | ACACAGACACAGAAACCGCC |
| IGF2BP2 Reverse | AACTGATGCCCGCTTAGCTT |
| DHX9 Forward | TCCAACTGGAATCCTTGGAC |
| DHX9 Reverse | TTTTCCCACATCCAGTAGCC |
| E-cad Forward | GACAACAAGCCCGAATT |
| E-cad Forward | GGAAACTCTCTCGGTCCA |
| N-cad Forward | CGGGTAATCCTCCCAAATCA |
| N-cad Reverse | CTTTATCCCGGCGTTTCATC |
| β-catenin Forward | TGCCAAGTGGGTGGTATAGAGG |
| β-catenin Reverse | CGCTGGGTATCCTGATGTGC |
| HMGA1 Forward | AGCGAAGTGCCAACACCTAAG |
| HMGA1 Reverse | TGGTGGTTTTCCGGGTCTTG |
| HMGA1 3UTR Forward | CAGCTTCCTTCTGGGACTGG |
| HMGA1 3UTR Reverse | GTGTAGTGTGGTGGTGAGGG; |
| GAPDH Forward | AAGGTCGGAGTCAACGGATTTG |
| GAPDH Reverse | CCATGGGTGGAATCATATTGGAA |
| 18S rRNA Forward | GTAACCCGTTGAACCCCATT |
| 18S rRNA Reverse | CCATCCAATCGGTAGTAGCG |
| β actin Forward | TCGTGCGTGACATTAAGGAG |
| β actin Reverse | ATGCCAGGGTACATGGTGGT |
