# Supplementary figures and images for: LINC00460/DHX9/IGF2BP2 complex promotes colorectal cancer proliferation and metastasis by mediating HMGA1 mRNA stability depending on m6A modification
Source: J Exp Clin Cancer Res. 2021 Feb 1;40:52. doi: 10.1186/s13046-021-01857-2 (PMC7851923; doi:10.1186/s13046-021-01857-2)

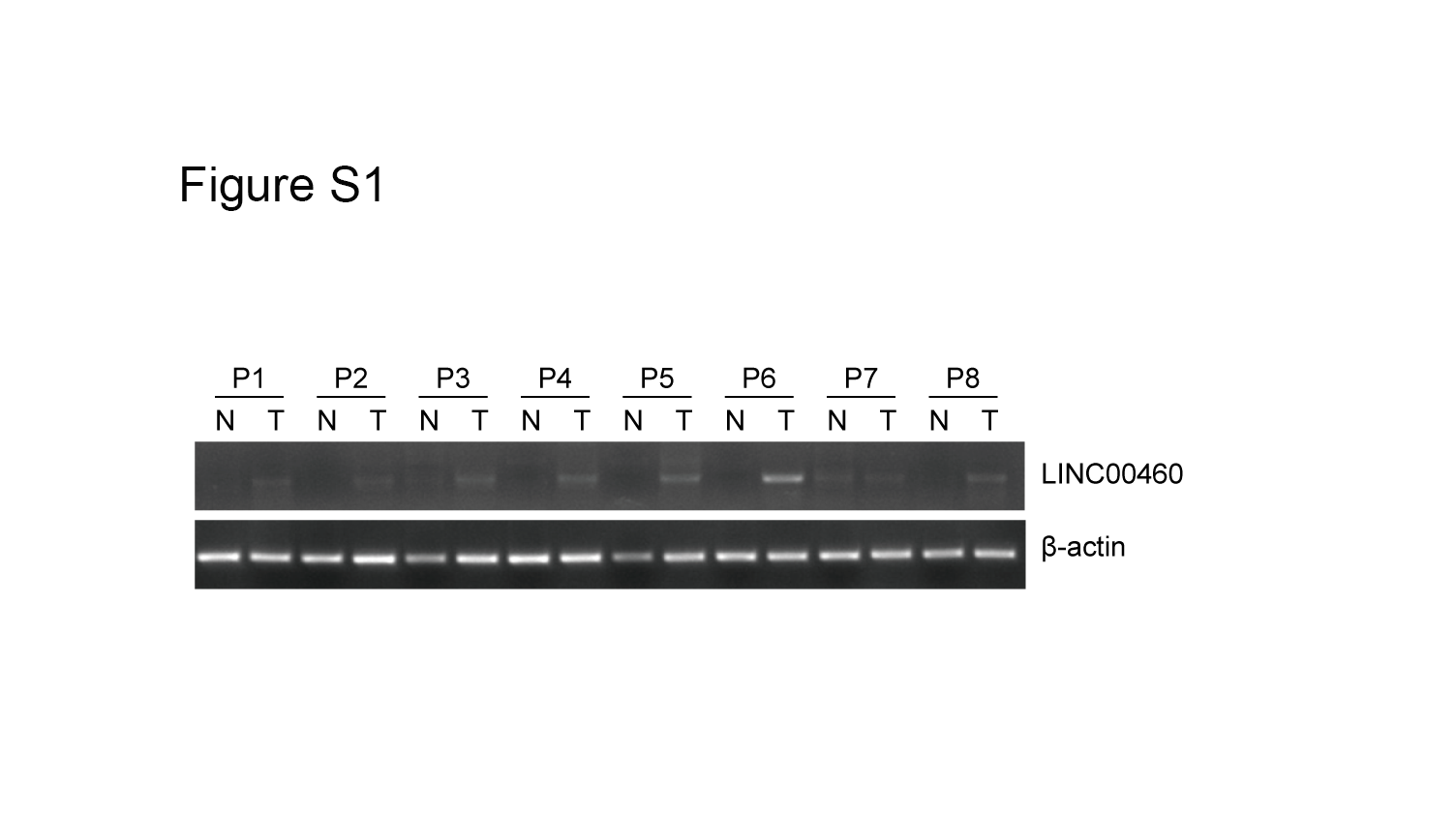

Supplement: Supplementary file 3 — Additional file 3: Figure S1. PCR detection of LINC00460 in CRC tissues and corresponding adjacent normal colon tissues using gel electrophoresis [file 13046_2021_1857_MOESM3_ESM.tif]

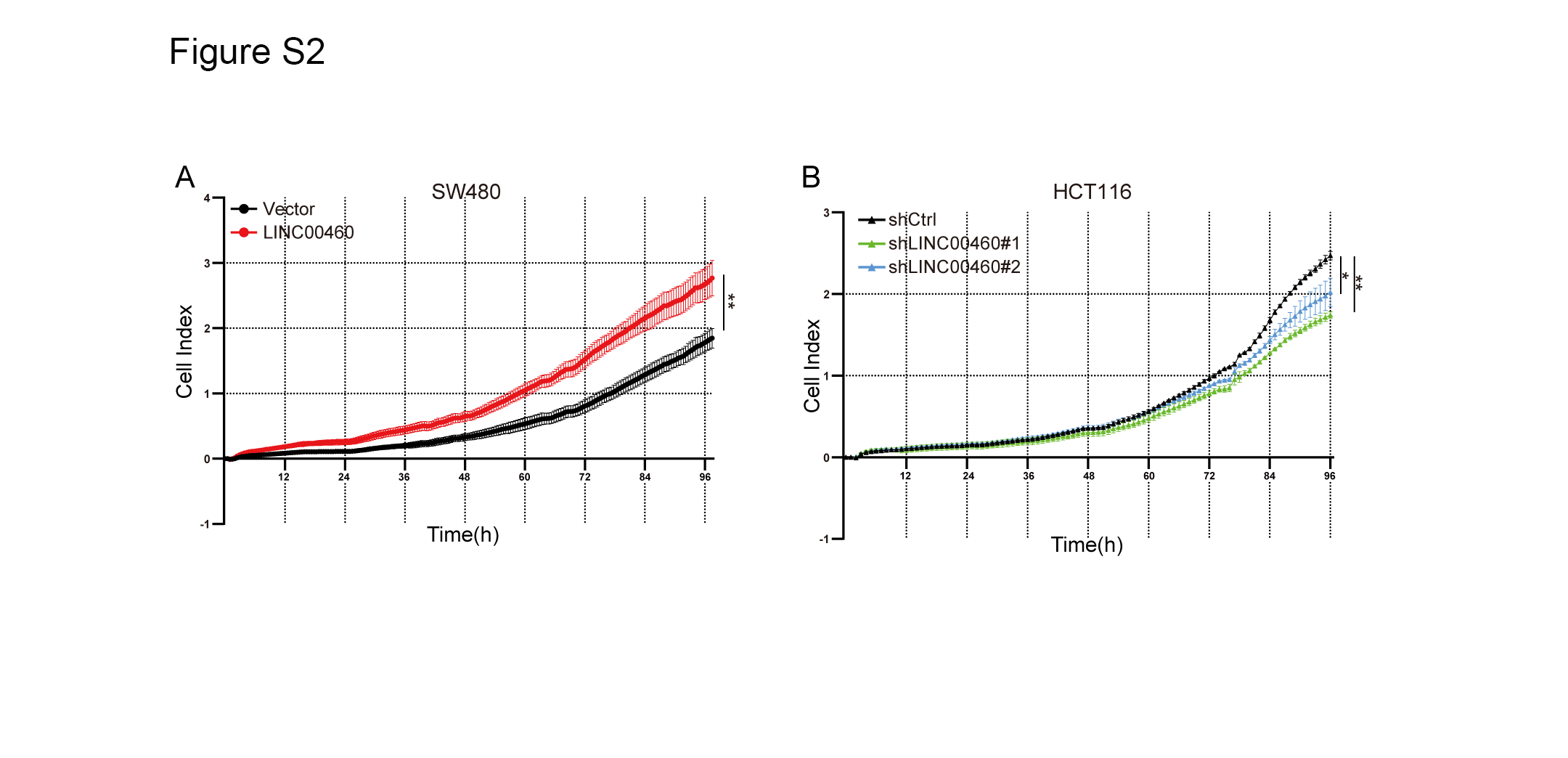

Supplement: Supplementary file 6 — Additional file 6: Figure S2. RTCA assays showed LINC00460 positively regulated CRC cells proliferation. (A, B) Effect of LINC00460 KD or OE on HCT116 and SW480 cells proliferation as assessed by Real-Time Cell Analyzer (RTCA, XCELLigence) assays (*p < 0.05, **p < 0.01, ***p < 0.001). [file 13046_2021_1857_MOESM6_ESM.tif]

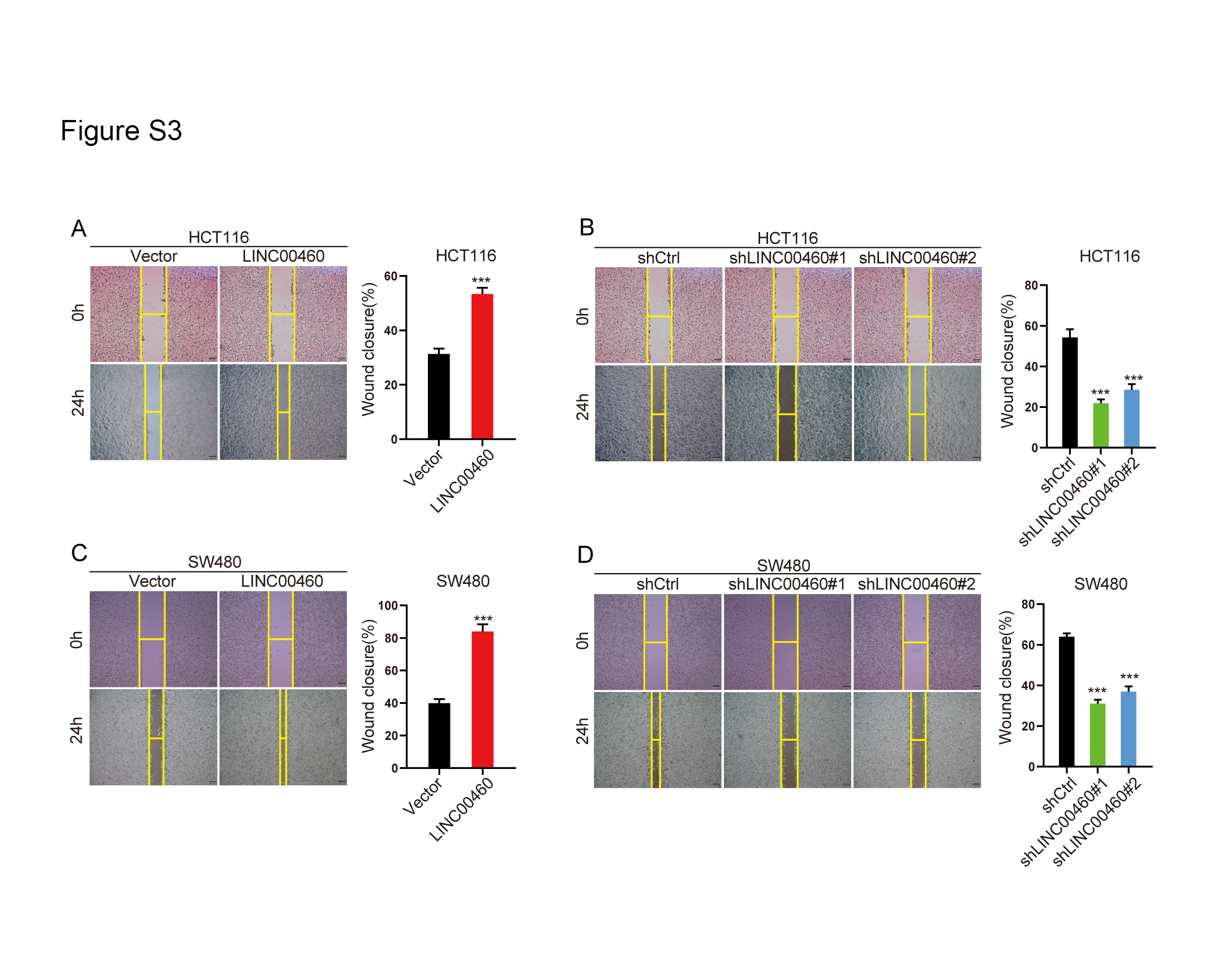

Supplement: Supplementary file 7 — Additional file 7: Figure S3. Wound-healing assays showed LINC00460 positively regulated CRC cell mobility. (A, B, C, D) The ability of motility in HCT116 and SW480 cells ± LINC00460 KD/OE tested by wound-healing assay. Statistical analysis was performed using unpaired t-tests. All statistical tests were two-sided. *p < 0.05, **p < 0.01, ***p < 0.001. [file 13046_2021_1857_MOESM7_ESM.tif]

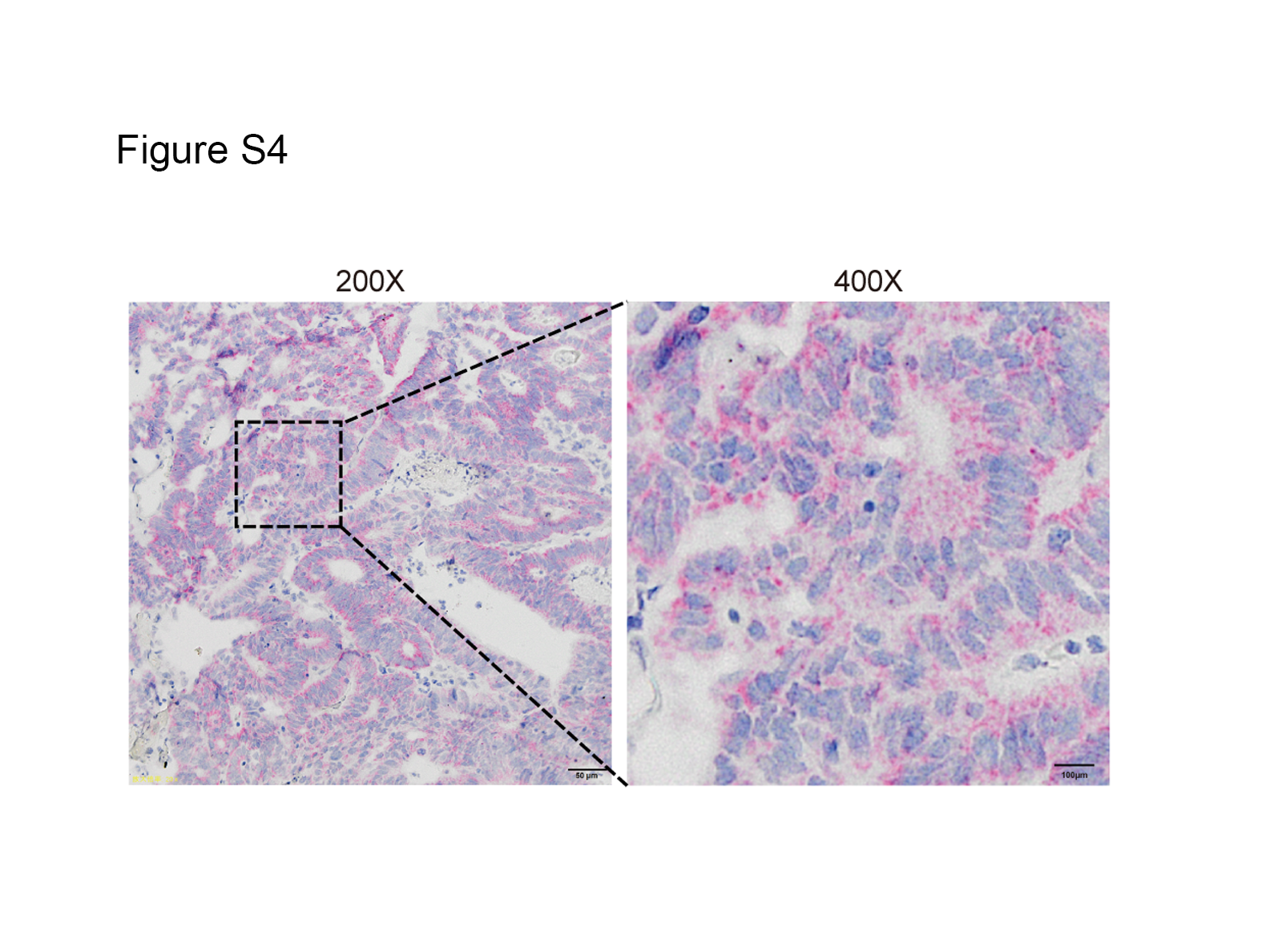

Supplement: Supplementary file 8 — Additional file 8: Figure S4. LINC00460 intracellular localization in CRC tissues. [file 13046_2021_1857_MOESM8_ESM.tif]

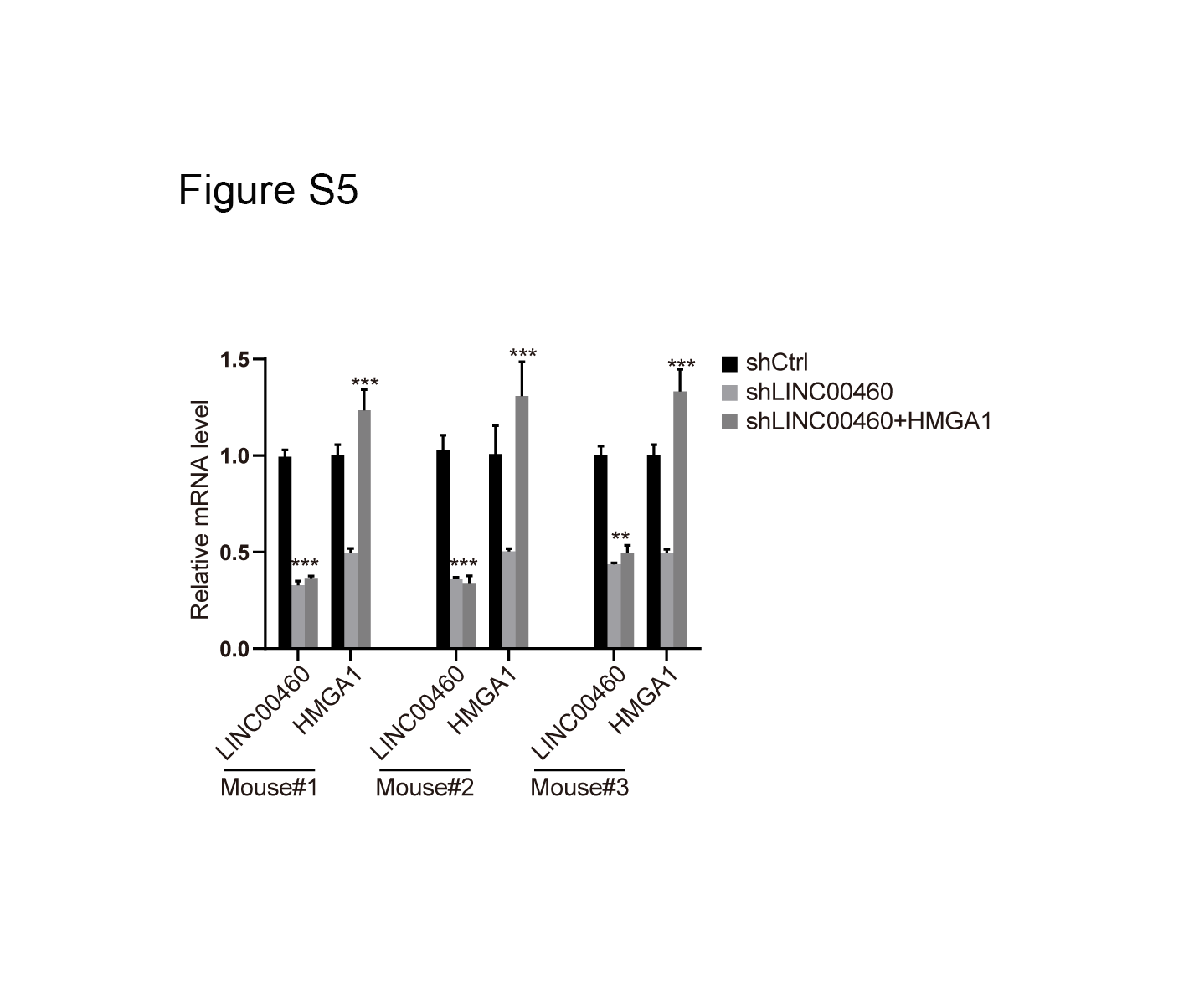

Supplement: Supplementary file 9 — Additional file 9: Figure S5. Relative mRNA expression levels of HMGA1 expression in tumor xenografts. [file 13046_2021_1857_MOESM9_ESM.tif]

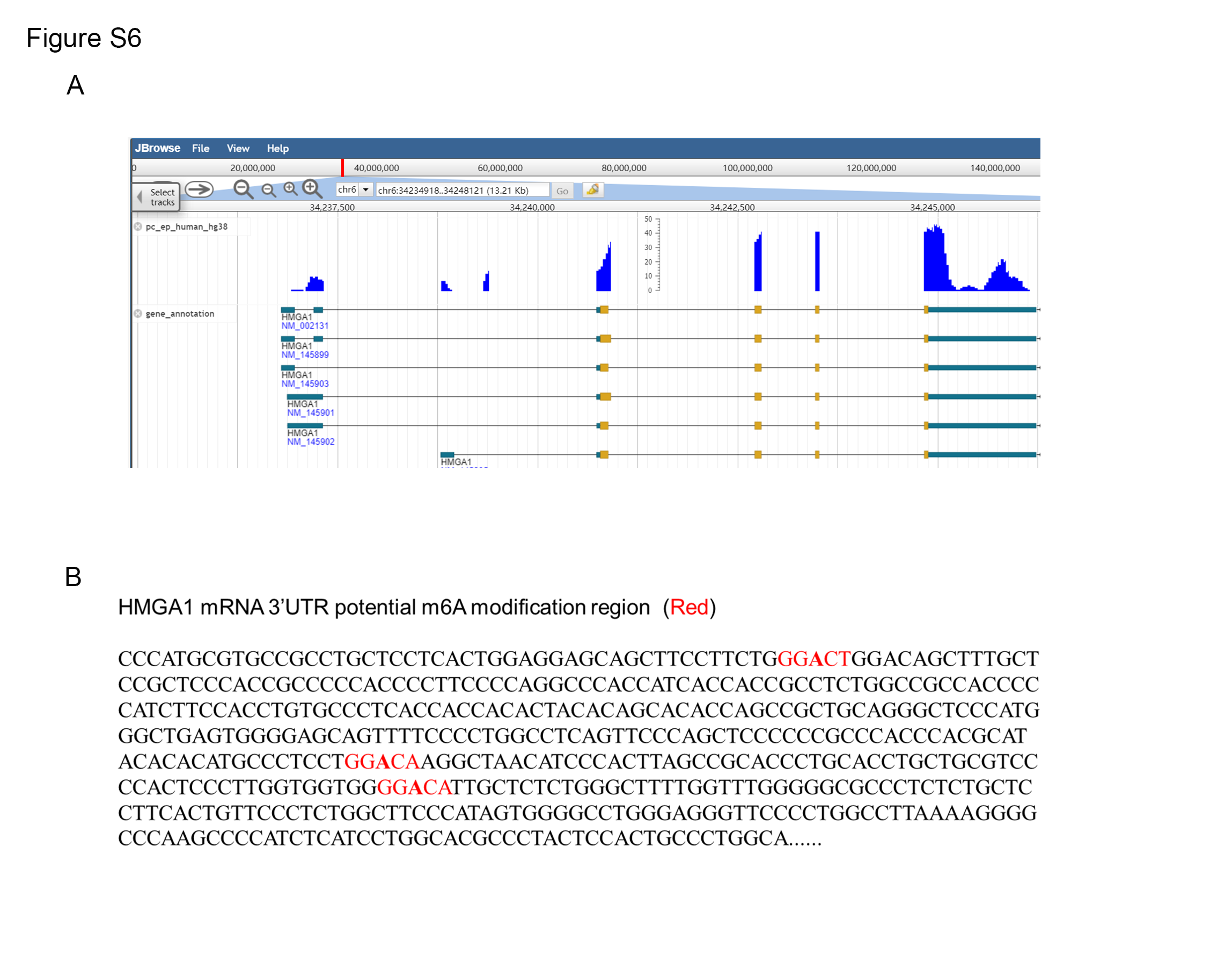

Supplement: Supplementary file 10 — Additional file 10: Figure S6. Prediction of HMGA1 mRNA m6A modification status. (A) HMGA1 mRNA m6A modification status in the MethylTranscriptome DataBase v2.0. (B) Potential METTL3 modified regions in the 3’UTR of HMGA1 mRNA. [file 13046_2021_1857_MOESM10_ESM.tif]
