# Supplementary material for: LINC00460/DHX9/IGF2BP2 complex promotes colorectal cancer proliferation and metastasis by mediating HMGA1 mRNA stability depending on m6A modification
Source: J Exp Clin Cancer Res. 2021 Feb 1;40:52. doi: 10.1186/s13046-021-01857-2 (PMC7851923; doi:10.1186/s13046-021-01857-2)
Supplement: Supplementary file 4 — Additional file 4: Table S2. Univariate Cox regression analysis of LINC00460 expression and clinicopathologic variables predicting the survival of CRC patients [file 13046_2021_1857_MOESM4_ESM.docx]

**Table S2** Univariate Cox regression analysis of LINC00460 expression and clinicopathologic variables predicting the survival of CRC patients

| **Variables*** | **Overall Survival** | | **Disease Free Survival** | |
| --- | --- | --- | --- | --- |
|  | **HR (95%CI)** | ***P*** | **HR (95%CI)** | ***P*** |
| LINC00460 | 2.526 (1.696–3.761) | ＜0.001 | 3.437 (1.323–8.927) | 0.001 |
| Age | 1.371 (0.985–1.907) | 0.061 | 2.615(1.130–6.054) | 0.025 |
| Gender | 1.357 (0.990–1.859) | 0.057 | 2.030 (1.009–4.084) | 0.047 |
| Tumor diameter | 1.747(1.267–2.409) | 0.001 | 2.321 (1.134–4.754) | 0.021 |
| Differentiation | 0.662(0.414–1.061) | 0.087 | 0.455 (0.138–1.500) | 0.196 |
| Depth of invasion | 1.816 (1.146–2.878) | 0.011 | 4.830 (1.154–20.216) | 0.031 |
| TNM stage | 2.642 (1.904–3.666) | ＜0.001 | 4.793 (2.217–10.362) | ＜0.001 |
| LNM | 1.959 (1.431–2.683) | ＜0.001 | 3.145 (1.552–6.373) | 0.001 |
| Metastasis | 4.600 (2.914–7.262) | ＜0.001 |  |  |

Abbreviations: HR: Hazard Ratio; CI: Confidence Interval; LNM: Lymph Node Metastasis.

Variables*: LINC00460: Low *vs* High; Age: ≤60(years) *vs*＞60(years); Gender: Male *vs* Female; Tumor diameter: ≤4.5(cm) *vs*＞4.5(cm); Differentiation: Poor *vs* Moderate/High; Depth of invasion: T1/T2 *vs* T3/T4; TNM stage: I/II *vs* III/IV; LNM: N0 *vs* N1/N2/N3; Metastasis: M0 *vs* M1.
