# Supplementary material for: LINC00460/DHX9/IGF2BP2 complex promotes colorectal cancer proliferation and metastasis by mediating HMGA1 mRNA stability depending on m6A modification
Source: J Exp Clin Cancer Res. 2021 Feb 1;40:52. doi: 10.1186/s13046-021-01857-2 (PMC7851923; doi:10.1186/s13046-021-01857-2)
Supplement: Supplementary file 5 — Additional file 5: Table S3. Multvariate Cox regression analysis of LINC00460 expression and clinicopathologic variables predicting the survival of CRC patients [file 13046_2021_1857_MOESM5_ESM.docx]

**Table S3** Multivariate Cox regression analysis of LINC00460 expression and clinicopathologic variables predicting the survival of CRC patients

| **Variables*** | **Overall Survival** | | **Disease Free Survival** | |
| --- | --- | --- | --- | --- |
|  | **HR (95%CI)** | ***P*** | **HR (95%CI)** | ***P*** |
| LINC00460 | 2.299 (1.539-3.435) | <0.001 | 2.751 (1.052-7.198) | 0.009 |
| Age | 1.288 (0.923-1.797) | 0.136 | 2.484 (1.761-3.502) | 0.051 |
| Differentiation | 0.743(0.460-1.198) | 0.223 | 0.624 (0.185-2.106) | 0.447 |
| Depth of invasion | 1.545 (0.964-2.475) | 0.071 | 2.863 (0.669-12.240) | 0.156 |
| TNM stage | 2.861 (1.774-4.614) | <0.001 | 6.244 (1.911-20.404) | 0.002 |
| LNM | 1.296 (0.814-2.063) | 0.275 | 2.484 (1.761-3.502) | 0.275 |

Abbreviations: HR: Hazard Ratio; CI: Confidence Interval; LNM: Lymph Node Metastasis.

Variables*: LINC00460: Low *vs* High; Age: ≤60(years) *vs*＞60(years); Differentiation: Poor *vs* Moderate/High; Depth of invasion: T1/T2 *vs* T3/T4; TNM stage: I/II *vs* III/IV; LNM: N0 *vs* N1/N2/N3.
